# A Neuromedin U Receptor Acts with the Sensory System to Modulate Food Type-Dependent Effects on *C. elegans* Lifespan

**DOI:** 10.1371/journal.pbio.1000376

**Published:** 2010-05-25

**Authors:** Wolfgang Maier, Bakhtiyor Adilov, Martin Regenass, Joy Alcedo

**Affiliations:** Friedrich Miescher Institute for Biomedical Research, Basel, Switzerland; Brown University, United States of America

## Abstract

The type of food source has previously been shown to be as important as the level of food intake in influencing lifespan. Here we report that different *Escherichia coli* food sources alter *Caenorhabditis elegans* lifespan. These effects are modulated by different subsets of sensory neurons, which act with *nmur-1*, a homolog of mammalian neuromedin U receptors. Wild-type *nmur-1*, which is expressed in the somatic gonad, sensory neurons, and interneurons, shortens lifespan only on specific *E. coli* food sources—an effect that is dependent on the type of *E. coli* lipopolysaccharide structure. Moreover, the food type-dependent effect of *nmur-1* on lifespan is different from that of food-level restriction. Together our data suggest that *nmur-1* processes information from specific food cues to influence lifespan and other aspects of physiology.

## Abstract 2

Different food types modulate worm lifespan and involve the neuropeptide receptor NMUR-1, which acts with the sensory neurons in a bacterial lipopolysaccaharide structure-dependent manner.

## Author Summary

Work on the model organisms *C. elegans* and *D. melanogaster* has contributed important and often surprising insights into the factors that determine lifespan. One intriguing finding is that lifespan in both animals can be extended or shortened by interfering with the function of neurons that smell or taste food. Indeed, specific taste neurons in *C. elegans* are required for the lifespan extension due to the restriction of the animals' level of food intake, while certain olfactory neurons in *Drosophila* inhibit this effect. Here we provide evidence that the sensory system also alters lifespan in response to specific food types as opposed to different food levels. *C. elegans* that feed on different *E. coli* strains can have different lifespans, which is not only dependent on the activities of a subset of sensory neurons but can also occur independently of food level restriction. We also show that the neuropeptide receptor NMUR-1 acts with the sensory system to affect lifespan in a manner dependent on the bacterial lipopolysaccharide structure. Thus, we identify both a food-derived factor and a component of a signaling pathway involved in the food-type effects on worm lifespan.

## Introduction

The sensory systems of *Caenorhabditis elegans* and *Drosophila melanogaster* have been shown to modulate the lifespan of these animals [1]–[4]. This sensory influence involves subsets of gustatory and olfactory neurons [2],[3] that either shorten or lengthen lifespan, which suggests that (i) some of the cues that affect lifespan are food-derived and that (ii) these cues can exert different effects on lifespan. Since a reduction in food levels can increase lifespan [5], it is possible that the sensory system influences lifespan by simply regulating the animal's general food intake, and, indeed, the sensory system has been implicated in the lifespan effects of food-level restriction in *Drosophila* [3]. On the other hand, the sensory influence on lifespan, at least in *C. elegans*, can be uncoupled from the sensory effects on feeding rate, development, and reproduction [1],[2]. Since the lifespan effect of food-level restriction has been linked to changes in feeding rates and decreased development and reproduction [5], this suggests that the sensory system also affects lifespan through other mechanism(s).

The *C. elegans* hermaphrodite has 60 sensory neurons with dendrites that terminate in ciliated endings [6]. These specialized structures contain dedicated sensory receptors [7],[8] and are thus the sites of recognition for different types of environmental cues, including gustatory, olfactory, thermal, and mechanical stimuli [9]. Within its natural environment, *C. elegans* encounters various types of bacteria that can serve as food sources. Similar to the sensory influence on lifespan, some of these food sources have been shown to alter lifespan independently of development and reproduction [10]. At the same time, not all but only a subset of food-sensing neurons influence the lifespan of *C. elegans* grown on the standard laboratory food source [2], *Escherichia coli* OP50 [11]. Together these data raise the possibility that sensory neurons promote the lifespan effects of different food sources through a mechanism distinct from that of food-level restriction.

In this study, we have investigated the role of the sensory system in the food-source influence on *C. elegans* lifespan and the signaling pathway(s) that might be involved in this process. We show that the *C. elegans* sensory system recognizes food types to affect longevity. We also identify (i) the neuromedin U receptor *nmur-1* as a neuropeptide signaling pathway involved in this process and (ii) a food-derived cue, the *E. coli* lipopolysaccharide (LPS) structure, which elicits the *nmur-1* response.

## Results

### The Sensory System Alters the Effects of Food Types on Lifespan

**Figure 1:**
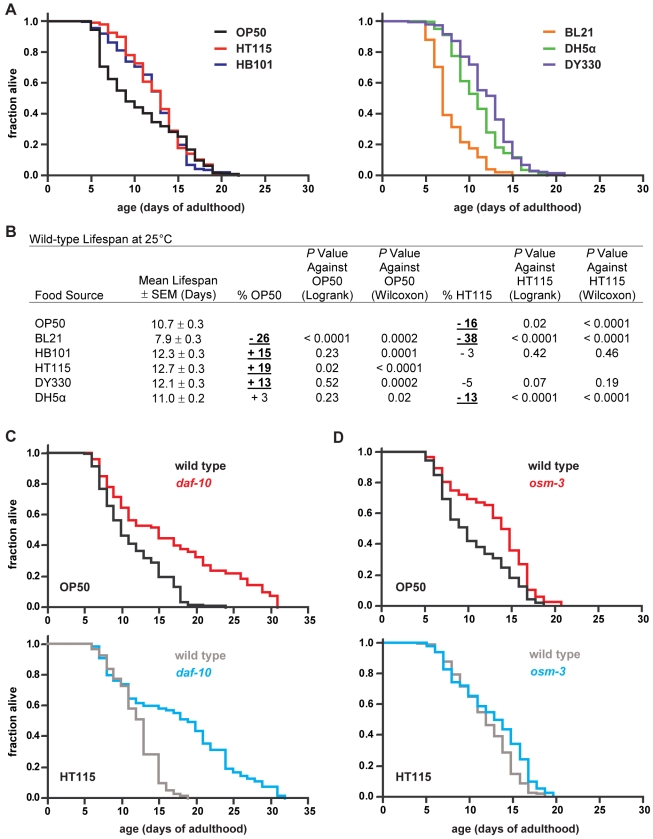
Sensory neurons modulate the effects of different types of food sources on lifespan. (A) Wild-type survival plots on different *E. coli* food sources and (B) statistics for cumulative data from two to three independent trials at 25°C (see Table S3 for statistics on individual trials). The % OP50 and % HT115 in (B) refer to mean lifespan changes relative to the two standard food sources. Bold underlined values indicate a significant difference in survival (Wilcoxon *p*≤0.01) on a given food source compared to either OP50 or HT115. Logrank test results are given for comparison (see Materials and Methods). (C) The lifespan of *daf-10(m79)* sensory mutants compared to wild type on two different *E. coli* food sources. The curves in this and subsequent panels represent cumulative data. Detailed data on these and subsequent survival analyses can be found in Tables 1 and/or S3. (D) The lifespan of wild type and *osm-3(n1540)* sensory mutants on different *E. coli* strains.

Wild-type *C. elegans* have altered lifespan on different *E. coli* strains (Figure 1A and 1B). Indeed, we found that at 25°C the mean lifespan of wild-type worms is shorter on OP50 than on HT115 (Figure 1A and 1B), another food source that is widely used [12],[13]. To test the hypothesis that sensory perception contributes to these food source-dependent effects, we measured the lifespan of sensory mutants on OP50 and HT115 at this temperature.

The gene *daf-10* encodes an ortholog of an intraflagellar transport complex protein that is required for cilia formation in a subset of sensory neurons (Table S1) [14],[15]. We observed that the lifespan of *daf-10* mutants is extended to the same extent (44% versus 46%; Table 1) compared to that of wild type when grown on either OP50 or HT115 (Figure 1C), which suggests that some sensory neurons shorten lifespan independently of these two food sources.

**Table 1:** Cumulative adult lifespans at 25°C.

Strain/Treatment	Mean Lifespan ± SEM (Days)	75th Percentile (Days)	Number of Animals Observed/Total Initial Animals	% Wild Type	*p* Value Against Wild Type (Logrank)	*p* Value Against Wild Type (Wilcoxon)	% of Specified Groups	*p* Value Against Specified Groups (Logrank)	*p* Value Against Specified Groups (Wilcoxon)
**Sensory Mutants**									
OP50: Wild type	11.3±0.4	15	130/150 (2)						
OP50: *daf-10(m79)*	16.3±0.9	22	78/148 (2)	**+44**	<0.0001	<0.0001			
HT115: Wild type	12.1±0.3	15	124/140 (2)				+7	0.94^a^	0.02^a^
HT115: *daf-10(m79)*	17.7±0.8	24	101/140 (2)	**+46**	<0.0001	<0.0001	+9	0.29^a^	0.13^a^
OP50: Wild type	10.5±0.3	14	187/220 (3)						
OP50: *osm-3(n1540)*	12.9±0.3	17	192/250 (3)	**+23**	<0.0001	<0.0001			
HT115: Wild type	11.8±0.2	15	198/220 (3)				**+12**	0.10^a^	0.0002^a^
HT115: *osm-3(n1540)*	12.6±0.3	16	219/250 (3)	*+7*	<0.0001	0.02	−2	0.37^a^	0.49^a^
***nmur-1*** ** Food-Dependence**									
OP50: Wild type	10.7±0.3	16	240/290 (4)						
OP50: *nmur-1*	15.0±0.3	18	230/292 (4)	**+40**	<0.0001	<0.0001			
BL21: Wild type	7.9±0.3	9	52/150 (2)				**−26**	<0.0001^a^	0.0002^a^
BL21: *nmur-1*	10.1±0.5	14	85/150 (2)	**+28**	0.0001	0.002	**−33**	<0.0001^a^	<0.0001^a^
HB101: Wild type	12.3±0.3	15	193/220 (3)				**+15**	0.23^a^	<0.0001^a^
HB101: *nmur-1*	15.1±0.2	17	173/220 (3)	**+23**	<0.0001	<0.0001	+1	0.25^a^	0.38^a^
HT115: Wild type	12.7±0.3	15	186/220 (3)				**+19**	0.02^a^	<0.0001^a^
HT115: *nmur-1*	13.3±0.3	16	166/210 (3)	+5	0.06	0.08	**−11**	<0.0001^a^	<0.0001^a^
DY330: Wild type	12.1±0.3	14	137/150 (2)				**+13**	0.52^a^	0.0002^a^
DY330: *nmur-1*	12.8±0.3	15	126/150 (2)	+6	0.05	0.11	**−15**	<0.0001^a^	<0.0001^a^
DH5α: Wild type	11.0±0.2	13	196/220 (3)				+3	0.23^a^	0.02^a^
DH5α: *nmur-1*	13.2±0.2	16	197/220 (3)	**+20**	<0.0001	<0.0001	**−12**	<0.0001^a^	<0.0001^a^
**Rescue Experiments**									
Line 1									
OP50: *jxEx4*	10.1±0.4	14	69/83 (1)						
OP50: *nmur-1*; *jxEx12*	11.9±0.5	15	54/66 (1)	**+18**	0.01^b^	0.009^b^			
OP50: *nmur-1*; *jxEx4*	14.4±0.4	17	73/88 (1)	**+42**	<0.0001^b^	<0.0001^b^	**+21**	0.0003^c^	0.0001^c^
Line 2									
OP50: *jxEx4*	12.8±0.4	17	109/127 (2)						
OP50: *nmur-1*; *jxEx40*	14.6±0.5	19	89/152 (2)	**+14**	0.002^b^	0.01^b^			
OP50: *nmur-1*; *jxEx4*	17.4±0.4	21	120/144 (2)	**+36**	<0.0001^b^	<0.0001^b^	**+19**	<0.0001^c^	<0.0001^c^
HT115: *jxEx4*	11.2±0.3	13	128/152 (2)						
HT115: *nmur-1*; *jxEx40*	13.3±0.3	16	146/169 (2)	**+19**	<0.0001^b^	<0.0001^b^			
HT115: *nmur-1*; *jxEx4*	12.8±0.2	15	146/162 (2)	**+14**	<0.0001^b^	<0.0001^b^	*−4*	0.004^c^	0.18^c^
Line 2 and second set of controls									
OP50: *jxEx14*	12.5±0.5	15	69/80 (1)						
OP50: *nmur-1*; *jxEx40*	12.8±0.5	15	45/80 (1)	+2	0.75^b^	0.71^b^			
OP50: *nmur-1*; *jxEx14*	15.1±0.5	18	46/70 (1)	**+21**	0.002^b^	0.0003^b^	**+18**	0.0006^c^	0.0008^c^
***E. coli*** ** LPS-Dependence**									
OP50: Wild type	11.9±0.8	17	34/40 (1)						
OP50: *nmur-1*	17.5±0.5	19	31/40 (1)	**+47**	<0.0001	<0.0001			
CS180: Wild type	14.3±0.2	17	205/240 (3)						
CS180: *nmur-1*	15.1±0.2	17	202/240 (3)	+5	0.05	0.03			
CS2198: Wild type	13.2±0.3	15	141/160 (2)				**−8**	0.006^d^	0.001^d^
CS2198: *nmur-1*	15.8±0.3	19	134/161 (2)	**+20**	<0.0001	<0.0001	+3	0.05^d^	0.19^d^
CS2429: Wild type	13.3±0.2	16	213/240 (3)				**−7**	0.01^d^	0.0003^d^
CS2429: *nmur-1*	16.2±0.3	19	188/240 (3)	**+22**	<0.0001	<0.0001	**+7**	<0.0001^d^	0.002^d^
CS1861: Wild type	13.4±0.5	17	72/80 (1)				−3	0.99^d^	0.61^d^
CS1861: *nmur-1*	14.5±0.5	17	63/80 (1)	+8	0.17	0.12	−5	0.56^d^	0.51^d^
***daf-10*** ** Epistasis**									
OP50: Wild type	11.3±0.4	15	130/150 (2)						
OP50: *nmur-1*	15.4±0.4	18	126/150 (2)	**+36**	<0.0001	<0.0001			
OP50: *daf-10(m79)*	16.3±0.9	22	78/148 (2)	**+44**	<0.0001	<0.0001			
OP50: *daf-10*; *nmur-1*	21.1±0.8	26	75/150 (2)	**+87**	<0.0001	<0.0001	**+29**	0.002^e^	<0.0001^e^
HT115: Wild type	12.1±0.3	15	124/140 (2)						
HT115: *nmur-1*	12.2±0.4	15	55/70 (1)	+1	0.76	0.82			
HT115: *daf-10(m79)*	17.7±0.8	24	101/140 (2)	**+46**	<0.0001	<0.0001			
HT115: *daf-10*; *nmur-1*	21.8±1.0	25	32/70 (1)	**+80**	<0.0001	<0.0001	**+23**	0.02^e^	0.002^e^
***osm-3*** ** Epistasis**									
OP50: Wild type	10.5±0.3	14	187/220 (3)						
OP50: *nmur-1*	14.0±0.3	17	188/220 (3)	**+33**	<0.0001	<0.0001			
OP50: *osm-3(n1540)*	12.9±0.3	17	192/250 (3)	**+23**	<0.0001	<0.0001			
OP50: *osm-3*; *nmur-1*	15.2±0.2	18	220/250 (3)	**+45**	<0.0001	<0.0001	**+18**	<0.0001^f^	<0.0001^f^
							**+9**	0.005^g^	0.009^g^
HT115: Wild type	11.8±0.2	15	198/220 (3)						
HT115: *nmur-1*	12.7±0.2	15	182/220 (3)	**+8**	0.008	0.009			
HT115: *osm-3(n1540)*	12.6±0.3	16	219/250 (3)	*+7*	<0.0001	0.02			
HT115: *osm-3*; *nmur-1*	13.8±0.2	17	217/250 (3)	**+17**	<0.0001	<0.0001	**+10**	0.01^f^	0.002^f^
							**+9**	<0.0001^g^	0.001^g^
CS180: Wild type	13.8±0.2	16	143/160 (2)						
CS180: *nmur-1*	14.4±0.2	17	143/160 (2)	*+4*	0.002	0.03			
CS180: *osm-3(n1540)*	15.5±0.2	17	134/160 (2)	**+12**	<0.0001	<0.0001			
CS180: *osm-3*; *nmur-1*	15.7±0.2	17	136/160 (2)	**+14**	<0.0001	<0.0001	+1	0.24^f^	0.24^f^
							**+9**	<0.0001^g^	<0.0001^g^
CS2429: Wild type	12.8±0.3	16	152/160 (2)						
CS2429: *nmur-1*	14.2±0.3	17	147/160 (2)	**+11**	<0.0001	0.0007			
CS2429: *osm-3(n1540)*	15.3±0.3	18	140/160 (2)	**+20**	<0.0001	<0.0001			
CS2429: *osm-3*; *nmur-1*	14.8±0.3	18	148/160 (2)	**+16**	<0.0001	<0.0001	−3	0.32^f^	0.32^f^
							+4	0.02^g^	0.05^g^
***daf-2*** ** Epistasis on OP50**									
Wild type	11.6±0.5	15	62/70 (1)						
*nmur-1*	15.3±0.4	17	61/70 (1)	**+32**	<0.0001	<0.0001			
*daf-2(e1370)*	33.3±1.2	39	54/70 (1)	**+188**	<0.0001	<0.0001			
*daf-2(e1370)*; *nmur-1*	36.7±1.1	43	59/71 (1)	**+216**	<0.0001	<0.0001	+10	0.02^h^	0.05^h^
Wild type	11.8±0.5	15	76/80 (1)						
*nmur-1*	14.8±0.4	17	72/80 (1)	**+25**	<0.0001	<0.0001			
*daf-2(e1368)*	21.0±1.1	29	62/80 (1)	**+78**	<0.0001	<0.0001			
*daf-2(e1368)*; *nmur-1*	24.4±0.8	29	67/80 (1)	**+107**	<0.0001	<0.0001	+16	0.24^i^	0.06^i^
***daf-16*** ** Independence on OP50**									
Wild type	11.7±0.3	15	128/141 (2)						
*nmur-1*	16.6±0.5	19	57/70 (1)	**+42**	<0.0001	<0.0001			
*daf-16(mu86)*	9.5±0.3	13	125/140 (2)	**−19**	<0.0001	<0.0001			
*daf-16*; *nmur-1*	11.8±0.3	13	127/139 (2)	±0	0.30	0.99	**+24**	<0.0001^j^	<0.0001^j^
***aak-2*** ** Independence on OP50**									
Wild type	11.2±0.5	14	69/80 (1)						
*nmur-1*	14.8±0.3	17	110/130 (1)	**+32**	<0.0001	<0.0001			
*aak-2(ok524)*	10.8±0.5	13	66/80 (1)	−4	0.49	0.74			
*nmur-1 aak-2*	13.8±0.4	16	64/80 (1)	**+23**	0.007	0.0001	**+28**	0.0001^k^	<0.0001^k^
***hsf-1*** ** Independence on OP50**									
Wild type	11.0±0.3	14	102/140 (2)						
*nmur-1*	14.7±0.4	18	99/140 (2)	**+34**	<0.0001	<0.0001			
*hsf-1(sy441)*	7.3±0.1	8	248/624 (2)	**−34**	<0.0001	<0.0001			
*hsf-1*; *nmur-1*	8.2±0.2	10	102/744 (2)	**−25**	<0.0001	<0.0001	**+12**	<0.0001^m^	0.0004^m^
***pmk-1*** ** Independence on OP50**									
Wild type	12.4±0.3	16	197/240 (3)						
*nmur-1*	15.1±0.3	18	189/240 (3)	**+22**	<0.0001	<0.0001			
*pmk-1(km25)*	12.4±0.3	15	178/291 (3)	±0	0.62	1.0			
*pmk-1*; *nmur-1*	14.2±0.3	17	166/300 (3)	**+15**	0.02	<0.0001	**+15**	0.002^o^	<0.0001^o^

We assayed wild-type and mutant worms in parallel in independent trials (details shown in Table S3) and we show statistics from the cumulative experiments on different *E. coli* strains. The 75th percentile is the age when the fraction of worms alive in each group falls below 0.25. The first number in the fourth column is the number of worms observed as having died, while the second number gives the total number of worms in each experiment, including worms that were censored during the course of the assay. The numbers in parentheses in the fourth column indicate the number of trials performed. Worms that crawled off the plate, exploded, or bagged were censored at the time of the event, allowing these worms to be incorporated into the data set until the censor date and to avoid loss of information. Differences that are significant (*p*≤0.01) according to the Wilcoxon test, which in most cases are also significant according to the logrank test, are underlined and in boldface type. Differences that are significant only according to the logrank test are italicized. The % difference between wild type and mutants under different conditions is indicated in the fifth column. The % difference between certain groups of worms that are specified by the superscripted symbols is shown in the eighth column. The superscripted symbols indicate the following:

a compared to the cumulative data for the same genotype assayed on OP50;

b compared to *jxEx4[myo-3p::rfp]* or *jxEx14[myo-3p::rfp]* on the same food source;

c compared to the rescue line on the same food source;

d compared to the cumulative data for the same genotype assayed on CS180;

e compared with *daf-10(m79)* tested on the same food source;

f compared with *osm-3(n1540)* tested in parallel on the same food source;

g compared with *nmur-1(ok1387)* assayed in parallel on the same food source;

h compared with *daf-2(e1370)*;

i compared with *daf-2(e1368)*;

j compared to the cumulative data for *daf-16(mu86)*;

k compared with *aak-2(ok524)*;

m compared to the cumulative data for *hsf-1(sy441)*; and

o compared to the cumulative data for *pmk-1(km25)*;

In contrast, worms that carry mutations in *osm-3*, which encodes a kinesin motor protein required for cilia formation in a different subset of sensory neurons (Table S1) [16],[17], live long relative to wild type only when grown on OP50, but not when grown on HT115 (Figure 1D; Table 1). This implies that at least some of the *osm-3*-expressing neurons sense the lifespan-influencing difference(s) between these food sources.

### *nmur-1* Affects Lifespan in a Food Source-Dependent Manner

Since *osm-3* functions in cilia structure formation rather than in directly sensing or translating food-derived cues, we searched for non-structural genes that would act with the sensory system to regulate the food source-dependent effects on lifespan. Candidate genes would include those encoding sensory receptors and downstream signaling molecules, like neuropeptides and their receptors, which help transmit or modulate sensory information. Unlike individual sensory receptors specific for single cues, a single downstream factor may affect the integration of several cues, which would make the effects of this class of genes more readily detectable.

The *C. elegans* genome has more than 75 neuropeptide-like genes and more than 1,000 G-protein-coupled receptors, some of which function as neuropeptide receptors [9],[18]–[21]. We focused on a subset of these genes based on the availability of mutations and on the evidence that their homologs in other animals regulate feeding and metabolism [19],[21]–[25]. We compared the lifespan of the different mutants on OP50 and HT115.

**Figure 2:**
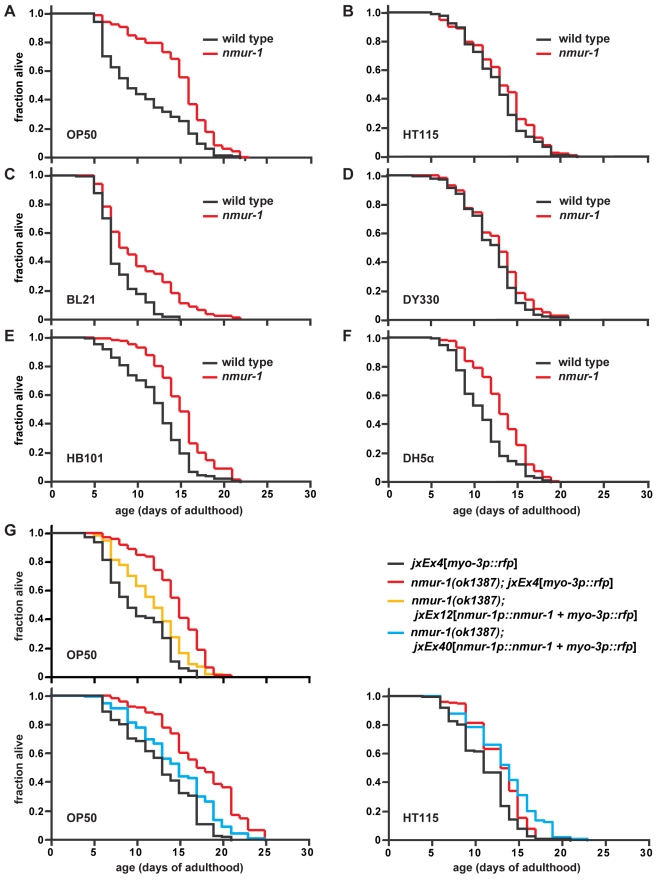
*nmur-1* modulates lifespan in a food source-dependent manner. (A–F) Lifespan of wild-type and *nmur-1(ok1387)* worms on different *E. coli* strains, which are indicated in the lower left corner of each panel. (G) The wild-type *nmur-1* genomic locus can rescue the food source-dependent long-life phenotype of *nmur-1(ok1387)* on OP50, without shortening lifespan on HT115. The lifespan of the two rescue lines, *nmur-1(ok1387)*; *jxEx12* and *nmur-1(ok1387)*; *jxEx40*, are compared to wild-type and *nmur-1* mutant worms that carry the *myo-3p::rfp* coinjection marker, *jxEx4*, alone.

While most neuropeptide signaling pathways had no effect on lifespan on the two food sources tested (Table S2), we found that animals carrying the deletion mutation *ok1387* within the gene *C48C5.1* live long on OP50 but not on HT115 (Figure 2A and 2B; Tables 1, S2, and S3). *C48C5.1* is predicted to encode a seven-transmembrane neuropeptide receptor (Figure S1) with homology to mammalian neuromedin U receptors (NMURs), whose peptide ligand, neuromedin U (NMU), has been shown to regulate food intake [24]. We renamed *C48C5.1* as *nmur-1*, since our study makes it the first phenotypically characterized member of the worm NMUR family, of which there are at least three other members—*nmur-2* (*K10B4.4*), *nmur-3* (*F02E8.2*), and *nmur-4* (*C30F12.6*). As a confirmation that the wild-type function of *nmur-1* is to shorten lifespan in a food source-dependent manner, we were able to rescue the long-life phenotype of the *nmur-1* mutation on OP50 with the wild-type *nmur-1* genomic locus, without shortening lifespan on HT115 (Figure 2G; Tables 1 and S3).

### *nmur-1* Acts with the Sensory System to Modulate the Food-Source Effects on Lifespan

**Figure 3:**
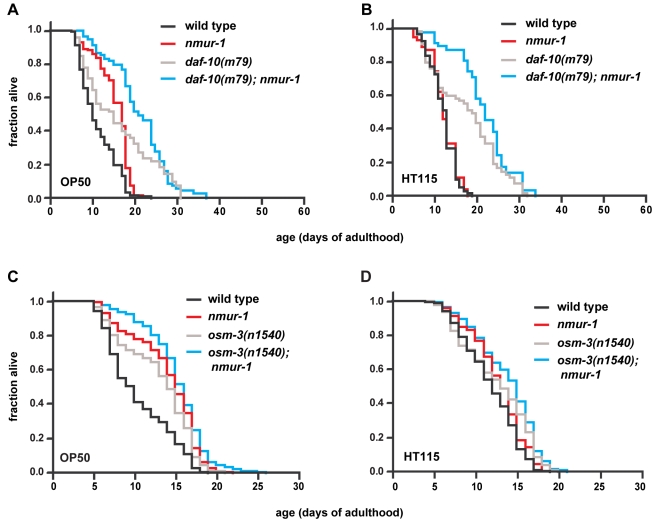
*nmur-1* acts with the sensory system to regulate lifespan. (A–B) The effects of *nmur-1* on the lifespan of *daf-10(m79)* mutants as measured on *E. coli* OP50 and HT115. (C–D) The effects of *nmur-1* on the lifespan of *osm-3(n1540)* mutants as compared on *E. coli* OP50 and HT115.

Next, we asked whether sensory neurons regulate the food source-dependent effects on lifespan through *nmur-1*. We found that loss of *nmur-1* still considerably increases the lifespan of *daf-10* sensory mutants on OP50 (Figure 3A; Table 1), which indicates that *nmur-1* acts in parallel at least to some *daf-10*-expressing neurons. Surprisingly, loss of *nmur-1* extends the lifespan of *daf-10* mutants also on HT115 (Figure 3B; Table 1), which may suggest that the lifespan of *nmur-1* mutants becomes food source-independent in the absence of *daf-10* activity. Thus, *nmur-1* appears to be subject not only to activation by certain environmental cues but also to inhibition by others.

In contrast, animals that carry both *nmur-1* and *osm-3* mutations have a lifespan phenotype similar to that of *nmur-1* single mutants on OP50 and HT115 (Figure 3C and 3D; Table 1). This suggests that *nmur-1* acts with *osm-3* either in a subset of *osm-3*-expressing sensory neurons or in downstream cells. We observed expression of a *gfp* reporter for *nmur-1* in the spermathecae of the somatic gonad, in several different types of sensory neurons, some of which co-express *osm-3* (Table S1) [16], and in interneurons (Table 2), some of which receive inputs from, or modulate the activity of, *osm-3*-expressing sensory neurons [6]. This expression pattern, together with the genetic interaction between the mutations in *nmur-1* and *osm-3*, suggests that *nmur-1* plays a role in the processing of sensory information derived by the worm from various food sources.

**Table 2:** *nmur-1p::gfp* expression at 25°C.

Cell/Tissue	Type	Function
ADFL/R	Amphid sensory neuron^a^	Chemosensation^b^, serotonergic neuron^c^
ADLL/R	Amphid sensory neuron^a^	Chemosensation^b^, nociception^d^
AFDL/R	Amphid sensory neuron^a^	Thermosensation^e^
OLQDL/R, OLQVL/R	Outer labial sensory neuron^a^	Mechanosensation^f^
AIAL/R (?)	Interneuron^a^	Integrates chemosensory information^a^^, ^ g
AIZL/R	Interneuron^a^	Integrates chemo-a ^, ^ g and thermosensory^e^ information
AVKL/R	Interneuron^a^	Unknown
DVA	Interneuron^a^	Stretch-receptor-mediated proprioception^h^
PVT	Interneuron^a^	Unknown
RICL/R	Interneuron^a^	Unknown
RIH	Interneuron^a^	Unknown
SDQL/R	Interneuron^a^	Unknown
PDA	Motor neuron^a^	Innervates posterior body wall muscles
ALA^a^	Neuron	Unknown
SIBDL/R^a^	Neuron	Unknown
SIBVL/R^a^	Neuron	Unknown
*And 2 other head neurons*		
Spermatheca	Somatic gonad	Reservoir for maturing spermatids and adult sperm

The superscripted symbols indicate the references that describe the types and known functions of the corresponding neurons;

a [6];

b [78];

c [83];

d [85];

e [82];

f [86];

g [87]; and

h [88]. The question mark indicates that the neuron expressing *nmur-1p::gfp* is likely to be AIA, since its position and morphology are consistent with those known for AIA, but this particular identification remains to be confirmed.

### The Effect of *nmur-1* on Lifespan Involves the *E. coli* LPS Structure

We then explored the possible differences between OP50 and HT115, which might be recognized by the worm. OP50 is derived from an *E. coli* B strain [11], whereas HT115 is from an *E. coli* K-12 strain [26],[27]. To determine whether *nmur-1* affects lifespan only on B strains but not on K-12 strains, we measured the lifespan of *nmur-1* mutants on other bacteria derived from these two lineages. Interestingly, we found that *nmur-1* mutants live long consistently on the B strain BL21 [28] and on HB101 (Figure 2C and 2E; Tables 1 and S3), a K-12 strain that contains a large stretch of B strain genomic DNA [29]. In contrast, the *nmur-1* long-life phenotype is absent on another K-12 strain, DY330 [30], and only occasionally present on the K-12 strain DH5α (Figure 2D and 2F; Tables 1 and S3) [31]. Together these data suggest that *nmur-1* affects lifespan in a largely B strain-dependent manner.

**Figure 4:**
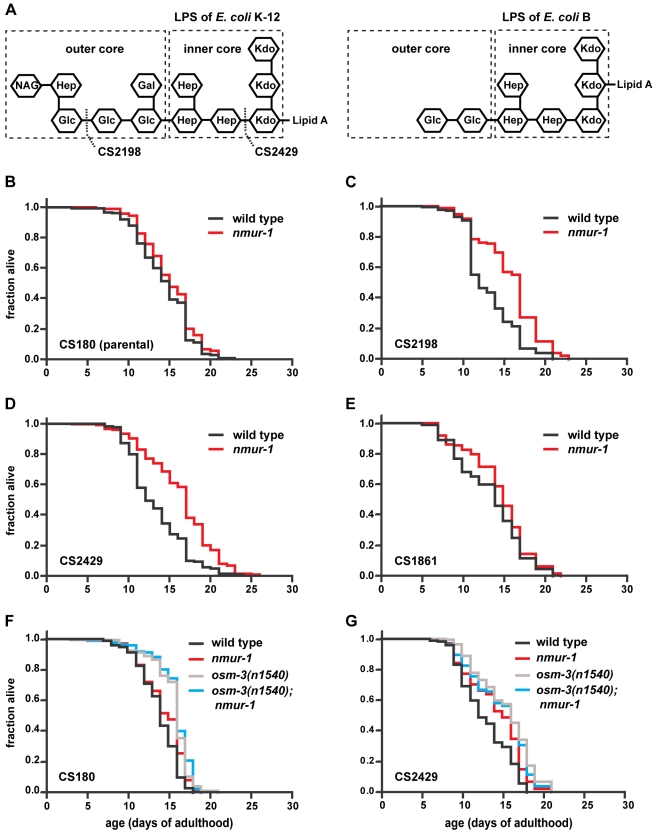
The *E. coli* LPS structure influences *C. elegans* lifespan in an *nmur-1*-dependent manner. (A) The LPS structures of *E. coli* K-12 and B strains have different sugar compositions [32],[33]. Strain CS180 expresses wild-type K-12 LPS. Strains CS2198 and CS2429 are isogenic derivatives of CS180 and express the indicated truncated forms of K-12 LPS. Strain CS1861 is derived from CS180 and expresses the *Shigella dysenteriae* 1 O Antigen attached to the tip of the full-length K-12 LPS. (B–E) Survival curves of wild-type and *nmur-1* mutant worms on CS180, CS2198, CS2429, and CS1861. (F–G) The lifespan of worms carrying mutations in *nmur-1* and/or *osm-3* as compared on *E. coli* strains with different LPS structures.

Although the B and K-12 strains clearly would have many differences, one of the few well-characterized molecular differences between these strains lies in the LPS structures (Figure 4A) on their outer membranes [32]–[34]. Since the LPS of the K-12 strain [33],[34] has a longer outer core than the LPS of the B strain [32], we tested whether LPS structure influences lifespan. We compared wild-type and *nmur-1* mutant worms on *E. coli* K-12 mutants that have truncated LPS to worms grown on the corresponding K-12 parent strain. We found that wild-type worms live shorter on the LPS truncation mutants CS2198 and CS2429 [35],[36] than on the isogenic parent strain CS180 (Tables 1 and S3), which expresses wild-type K-12 LPS [35]. On the other hand, *nmur-1* mutants live long compared to wild type only on the LPS truncation mutants (Figure 4C and 4D; Tables 1 and S3), but not on the K-12 parent strain (Figure 4B; Tables 1 and S3).

To exclude the possibility that all changes to the LPS will elicit the *nmur-1* response, we also measured the lifespan of worms grown on the K-12 strain CS1861 that expresses the *Shigella dysenteriae* 1 O Antigen fused to the end of the full-length K-12 LPS [36]. We observed no lifespan difference between wild type and *nmur-1* mutants on this strain (Figure 4E; Table 1). Together our data suggest that a short *E. coli* LPS structure can shorten worm lifespan in an *nmur-1*-dependent manner.

In contrast to *nmur-1*, we found that *osm-3* can affect lifespan independently of the *E. coli* LPS structure (Figure 4F and 4G; Table 1), which indicates that at least some of the *osm-3*-expressing neurons detect other food-derived cues. However, even on the CS180 and CS2429 bacterial food sources, *nmur-1* and *osm-3* appear to act together in influencing lifespan, since *osm-3*; *nmur-1* double mutants have the same lifespan phenotype as *osm-3* or *nmur-1* single mutants (Figure 4F and 4G; Table 1).

### The *nmur-1* Food-Source Effect on Lifespan Is Distinct from That of General Food-Level Restriction

**Figure 5:**
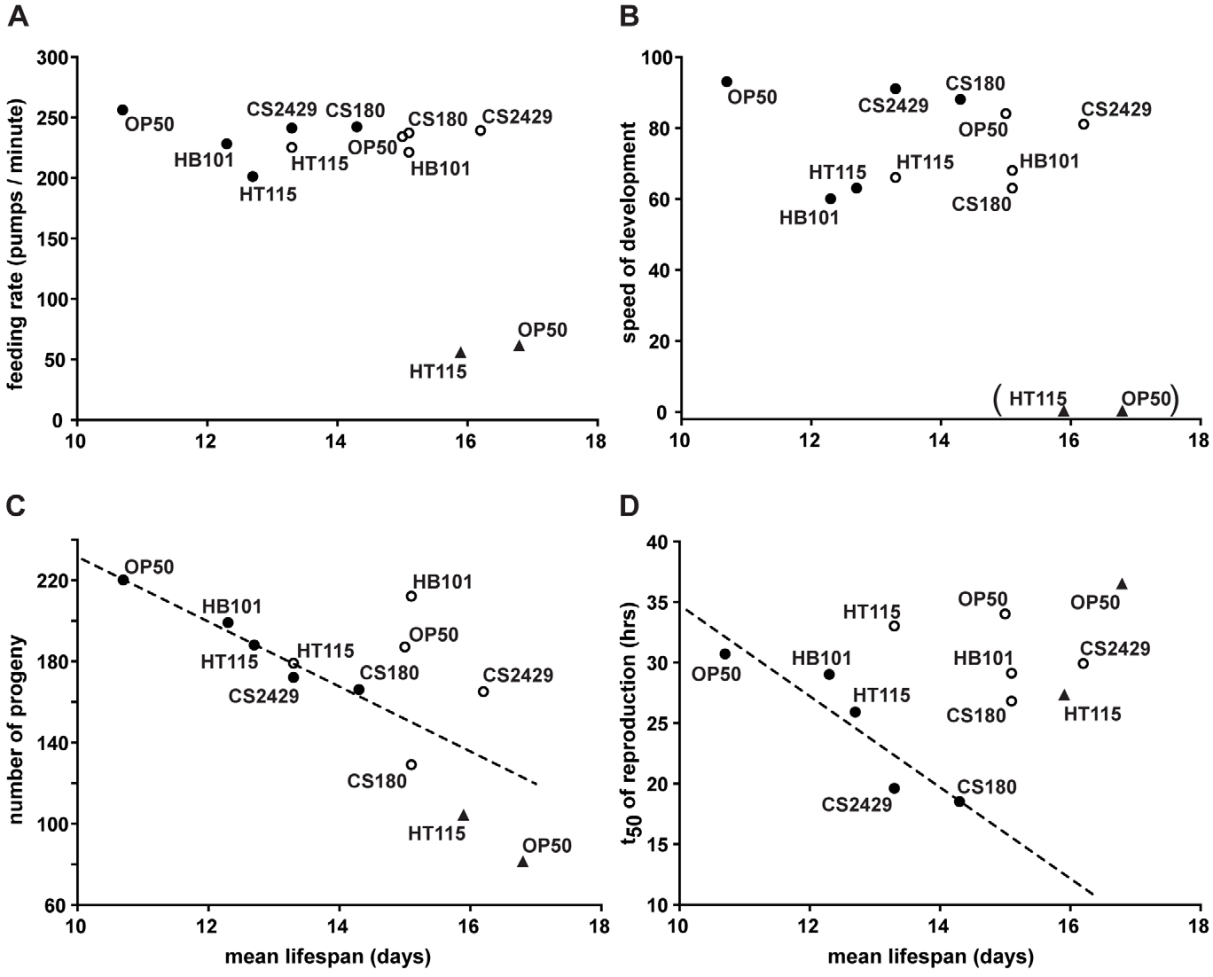
*nmur-1* exerts its effects on lifespan without inducing signs of food-level restriction. (A–D) The correlations of lifespan with feeding, development, and reproduction are shown across five different food sources. The figures are compiled from the lifespan data in Tables 1 and S3 and from the data on feeding rates, developmental rates, progeny numbers, and reproduction rates presented in Figures S2, S3, and S4. Pharyngeal pumping rates of young adults (A) and speed of development (B) do not correlate with mean lifespan for wild-type (closed circles) and *nmur-1* mutant worms (open circles), nor for the combined data, but are strongly reduced in food level-restricted *eat-2(ad1116)* mutants (closed triangles). The parentheses around the *eat-2* mutant data in (B) mean that the mutant speed of development falls outside the range of our index (see Materials and Methods). Progeny number (C) and t_50_ of reproduction (D) are inversely correlated with mean lifespan of wild-type worms (closed circles; *p* = 0.003 for total progeny and *p* = 0.029 for reproduction time). The dotted lines are the regression lines for total progeny and reproduction time (R^2^ = 0.960 and 0.837, respectively) calculated from the wild-type data alone. *nmur-1* (open circles) exerts an additional, reproduction-independent effect on lifespan, as suggested by the deviation from the regression lines of the corresponding data points for OP50, HB101, and CS2429.

Food type [37] and sensory neurons [3],[38] have been shown to mediate the lifespan extension induced by dietary restriction (DR), which is commonly studied through restriction of food levels. Thus, the food type-dependent effects on lifespan we observe might reflect different levels of DR experienced by wild-type and mutant worms on the various food sources. To address this possibility, we measured the feeding rates, speed of development, total progeny, and the rates of reproduction of wild-type and *nmur-1* mutant worms on five different *E. coli* strains (Figures S2 and S3), since restricting food levels is known to change these parameters [5]. For comparison, we used a genetic model for food-level restriction [39], a mutation in *eat-2* that impairs pharyngeal function [40], which leads to decreased feeding rates on both OP50 and HT115 (Figure S4A). Unlike the *nmur-1* mutation, we found that the *eat-2* mutation increases lifespan on both food sources (Figure S4C), which suggests that the food-type effects of *nmur-1* are not the same as those of food-level restriction. Moreover, we observed no correlation between lifespan and feeding rates or lifespan and development of wild-type or *nmur-1* mutant worms on the different food sources (Figures 5A, 5B, S2A, S2B, S2C, S2D, S3A, and S3B), which is also unlike the reported effects of restricting food levels [5].

As expected for a genetic model for food-level restriction, we found that the lifespan extension conferred by the *eat-2* mutation is accompanied by a decrease in total progeny on OP50 and HT115 (Figures 5C and S4B). Surprisingly, we also found that wild-type worms grown on different food sources do exhibit an inverse correlation between lifespan and number of progeny but that *nmur-1* mutants can still live long without a proportionate decrease in total progeny (Figures 5C, S2E, and S3C). This suggests that the food source-dependent effects on lifespan have reproduction-dependent and reproduction-independent components, the latter of which is uncovered by the *nmur-1* mutation. Interestingly, we also observed that food sources that increase wild-type lifespan induce the animals to reproduce faster (Figure 5D), which not only differs from *eat-2* mutants (Figure 5D) but is also the inverse of the effects shown for food-level restriction on rates of reproduction [5],[41]. At the same time, we again saw no correlation between the *nmur-1* mutant lifespan and its rates of reproduction on the different *E. coli* strains (Figures 5D, S2F, and S3D).

Since our data show that the effects of *eat-2* on *C. elegans* physiology differ from those of *nmur-1* or the different food sources, this suggests that the effects of the food sources and *nmur-1* on lifespan can be distinct from food-level restriction. Consistent with this idea, we observed that, unlike long-lived, food level-restricted animals that have decreased lipid storage [5], *nmur-1* mutants do not exhibit gross changes in fat storage compared to wild type on either OP50 or HT115 (Table S4).

### *nmur-1* Acts at Least Partly Independently of *daf-16* and *hsf-1*

**Figure 6:**
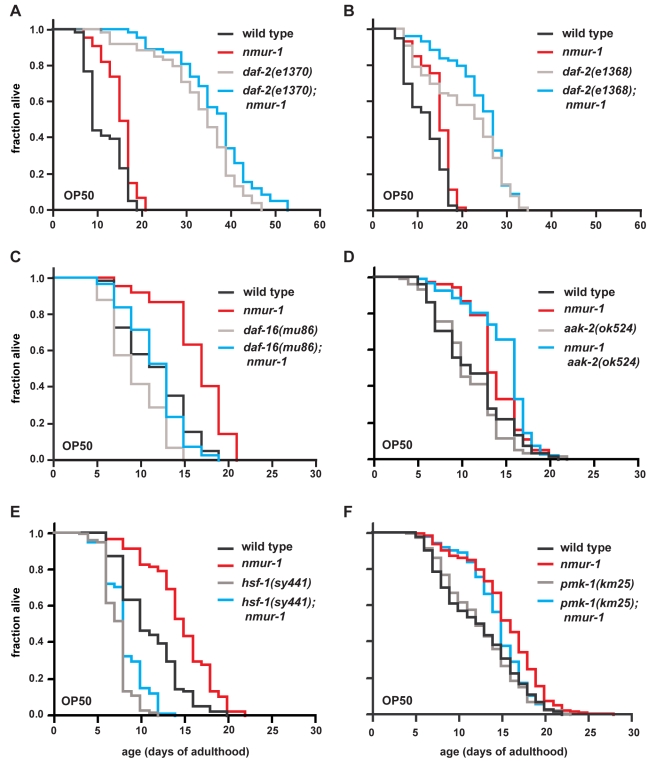
*nmur-1* acts at least partly independently of *daf-16* and *hsf-1*. (A–B) The effect of *nmur-1* on *daf-2* lifespan is shown for two different *daf-2* reduction-of-function mutant backgrounds. Since *daf-2* mutants undergo developmental arrest at 25°C, all strains in these experiments were grown at 20°C until the first day of adulthood, when the worms were shifted to 25°C to initiate lifespan measurements. (C) The effect of *nmur-1* on lifespan as assayed in a *daf-16(mu86)* null background. (D–F) The effect of *nmur-1* on the respective lifespan of *aak-2(ok524)*, *hsf-1(sy441)*, or *pmk-1(km25)* mutants.

Next, we asked whether *nmur-1* acts through the insulin/IGF-1 *daf-2* pathway [42], which has been shown to mediate a large part of the sensory influence on lifespan [1]. For example, the increased lifespan of *osm-3* sensory mutants on OP50 has been shown to be partly dependent on *daf-16* [1], a FOXO transcription factor that acts downstream of and is negatively regulated by *daf-2* [43]–[45]. Accordingly, we found that removing *nmur-1* does not significantly increase the lifespan of insulin/IGF-1 receptor *daf-2* mutant worms (Figure 6A and 6B; Table 1), but loss of *nmur-1* can still extend the lifespan of worms carrying a null mutation in *daf-16* (Figure 6C; Table 1). Thus, our data suggest that *nmur-1*, like at least some *osm-3*-expressing neurons, acts either with *daf-2* but at least partly independently of *daf-16*, or in parallel to the *daf-2*/*daf-16* pathway.

To identify other factors required for *nmur-1* to affect lifespan, we tested how removal of *nmur-1* would affect the short lifespan caused by mutations in genes proposed to act independently of *daf-16* [46]–[48]. We found that loss of *nmur-1* can still extend the lifespan of animals with a mutation in either (i) the AMP-dependent kinase *aak-2* (Figure 6D; Table 1), which regulates energy metabolism [46]; (ii) the heat shock transcription factor *hsf-1* (Figure 6E; Table 1), which regulates stress response [47],[49],[50]; or (iii) the p38 MAPK *pmk-1* (Figure 6F; Table 1), which regulates innate immunity [48],[51]. Although none of these factors appears essential for *nmur-1* function, we did observe partial suppression of the *nmur-1* phenotype in the *hsf-1* mutant background. This could suggest that *nmur-1* affects lifespan by acting through several parallel pathways that include *hsf-1* and/or *daf-16*.

## Discussion

Food is a complex environmental factor that affects many physiological processes, including lifespan. In the laboratory, *C. elegans* are grown on agar plates, on which the bacterial lawn that serves as the food source presumably provides a large part of the worm's chemosensory and mechanosensory inputs. Thus, the previous finding that some gustatory and olfactory neurons function either to shorten or lengthen *C. elegans* lifespan [2] makes it likely that food-derived cues affect longevity through the sensory system.

Some sensory neurons have been shown to be required for the prolonged lifespan conferred by DR [3],[38], i.e., under conditions of limited food availability. However, the fact that the sensory system also modulates lifespan when food is abundant suggests that the sensory influence on lifespan involves more than one mechanism, as illustrated in this and previous studies [1],[2].

### Sensory Neurons Recognize Food Types to Affect Lifespan

If food-derived cues alter lifespan through the sensory system, then it is likely that impairment of a specific set of sensory neurons that detect a given set of cues would affect lifespan only on some food sources. In this study, we provide a detailed investigation of the interdependence between food and sensory perception in regulating *C. elegans* longevity. We show not only that wild-type lifespan is modulated by different *E. coli* food sources (Figure 1A and 1B) but also that three genes, which have been shown to be expressed and/or act in sensory neurons, have food source-dependent effects on lifespan.

Mutations in two of these genes—*osm-3* and *nmur-1*—increase lifespan on OP50 but not on HT115 (Figures 1D and 2; Tables 1 and S3). Since the effects of these mutations are non-additive (Figure 3C and 3D; Table 1), this suggests that *osm-3 and nmur-1* influence lifespan through a common mechanism. On the other hand, a mutation in the third gene, *daf-10*, not only extends lifespan on both OP50 and HT115 (Figure 1C; Table 1) but also alters the food type-dependence of the *nmur-1* effect on lifespan (Figure 3A and 3B; Table 1).

Together with their requirement in the formation of the sensory cilia in subsets of neurons [15],[16], the *osm-3* and *daf-10* data are consistent with a role for sensory perception in the food source-dependent effects on lifespan. In addition, the identification of a neuropeptide receptor gene, *nmur-1*, that interacts with *osm-3* and *daf-10* (Figures 3, 4F and 4G; Table 1) suggests a mechanism through which the sensory system mediates the effects of specific food cues on lifespan. The *nmur-1* expression in sensory neurons and interneurons (Table 2) suggests that *nmur-1* modulates the transduction of signals downstream of the sensory receptors. Based on the observed interactions among these three genes, we propose the following model: (i) *osm-3*-expressing sensory neurons detect the presence of certain food-derived cues and transmit this information through an *nmur-1*-dependent pathway, and (ii) a different set of *daf-10*-expressing neurons detects other food cues, some of which inhibit *nmur-1* activity.

According to this model, the expression patterns (Table S1) of *osm-3* [16] and *daf-10* [15] should help define the candidate sensory neurons that might recognize the food cues that shorten or extend lifespan through *nmur-1*. *daf-10* is necessary for proper cilia morphology in the mechanosensory CEP neurons and some unidentified neurons in the head and tail sensory organs called the amphids and phasmids, respectively [15]. Several amphid neurons also express *osm-3* [16]: these include two pairs of gustatory neurons, ASI and ASG, that have been found to shorten lifespan on OP50 [2], and two other gustatory neuron pairs that co-express *nmur-1*—ADF, which by itself has no lifespan effect on OP50 [2], and ADL. In addition, *osm-3* is expressed outside of the amphid organs in the IL2 inner labial head neurons and in the phasmid tail neurons [16], all of which have been proposed to have chemosensory function [15],[52].

### A Neuropeptide Receptor of the NMUR Family Mediates the Sensory Influence on Lifespan

Our discovery of a food source-dependent function for the *C. elegans nmur-1* gene is consistent with the known food-associated activities of other members of the NMUR signaling pathway in mammals [24],[53] and insects [54],[55]. In mammals, NMUR2, the receptor isoform expressed in the central nervous system, and its ligand, the octapeptide NMU-8, have been implicated in the regulation of food intake and energy expenditure [24],[53]. In *Drosophila*, the gene *hugin* encodes two of the peptide ligands, PK-2 and HUG-γ, recognized by two of four NMUR isoforms [54]–[56]. *hugin* regulates not only the food-seeking behavior and feeding rate of larvae but also affects the rate of food intake of adult flies in a food type-dependent manner [54]. Like *hugin*, we find that *nmur-1* exerts food-type specific effects on feeding rate (Figure S2A and S2C), although the *nmur-1* regulation of this process appears to be parallel to its regulation of lifespan (Figure 5A). Similar to the neuronal expression of *nmur-1*, *Drosophila hugin* is expressed in interneurons that appear to relay gustatory information [54]. At present, a potential role for the fly or mammalian NMUR signaling pathways in the regulation of lifespan has not been reported. However, the evolutionary conservation of several aspects of NMUR signaling leads us to speculate that the effects on lifespan by this system might also be conserved across species.

The *Drosophila* NMU signaling system also includes a second neuropeptide precursor gene, *capability*, that encodes three other peptide ligands, CAPA-1, CAPA-2, and CAPA-3 (also called PK-1), that can activate three of the fly NMUR isoforms [56]. The *C. elegans* homolog of *capability*, *nlp-44*, has recently been identified [57]. Like *capability*, it is predicted to give rise to three peptides, one of which activates the receptor encoded by *nmur-2* [57]. A mutation of *nmur-2* gives no lifespan phenotype on the food sources we have tested (Table S2), but it will be interesting to determine whether peptides derived from *nlp-44* can also activate NMUR-1.

A role of *nmur-1* in the sensory influence on lifespan is supported by its expression in a number of sensory neurons and interneurons (Table 2). However, it remains possible that sensory cues regulate *nmur-1* activity at the level of the somatic gonad, which is the only non-neuronal tissue that expresses the *nmur-1* reporter gene (Table 2). At the same time, the expression of *nmur-1* in a relatively large number of cells also makes it likely that the parallel effects of *nmur-1* on lifespan, feeding rate, development, and reproduction (Figures 5A–5D, S2, and S3) are mediated by its activity in different subsets of cells.

The food source-dependent activities of *nmur-1* raise the possibility that other neuropeptide signaling pathways—many of which are associated with the sensory system [18]–[20],[25] —will also affect lifespan or other aspects of physiology only under specific conditions. Although most of the neuropeptide signaling pathways we have screened so far on two food sources show no effect on lifespan (Table S2), it remains possible that they will have effects on other food types. Thus, the large repertoire of neuropeptides and their receptors in *C. elegans* might serve to translate environmental complexity into appropriate physiological responses.

### The Bacterial LPS Represents a Food-Derived Cue that Influences Lifespan

We find that wild-type worms live shorter on the *E. coli* B strains BL21 and OP50 than on K-12 strains, like HT115 and DY330 (Figure 1A and 1B). Conversely, the *nmur-1* mutation causes reproducible lifespan extensions on the B strains but not on the K-12 strains (Figure 2; Tables 1 and S3). Since B and K-12 strains differ in their LPS structure, we have tested the lifespan effects of specific alterations in the K-12 LPS that mimic aspects of the B strain LPS (Figure 4A). Although the effect of LPS on wild-type lifespan is not large, wild-type worms do live longer on full-length than on truncated forms of the K-12 LPS (Tables 1 and S3). We also find that the *nmur-1* effect on lifespan is LPS-dependent and suppressed by full-length K-12 LPS but not by its truncated versions (Figure 4; Tables 1 and S3).

Although the LPS experiments were carried out in isogenic bacterial backgrounds, the effects of the LPS alterations might be indirect since they could lead to secondary changes in bacterial metabolism or surface structure. Indeed, LPS truncations have been shown to interfere with the expression of outer membrane proteins, increase capsule polysaccharide levels, and redistribute phospholipids from the inner to the outer leaflet of the outer membrane ([58] and references therein). However, these secondary changes have only been observed with mutations that disrupt the inner core of the LPS, like the mutation present in the CS2429 strain (Figure 4A), and thereby compromise the integrity of the outer membrane [58]. No such effects have been reported for truncations that affect only the LPS outer core, like the mutation in CS2198 (Figure 4A). Thus, the observation that *nmur-1* extends lifespan on both CS2429 and CS2198 argues for a direct effect of the bacterial LPS on worm lifespan. Direct recognition of LPS is biologically plausible: LPS is the predominant component of the outer membrane of gram-negative bacteria and is consequently used by multicellular organisms from diverse phyla to recognize bacteria in the context of defense against pathogens [59],[60].

Nevertheless, the LPS structure is clearly only one of potentially many food-derived cues that influence worm lifespan. This is most evident from the LPS-independent lifespan phenotype of *osm-3* mutants (Figure 4F and 4G; Table 1), and from the fact that the lifespan extension by the *nmur-1* mutation is greater on OP50 than on any other strain with a similar, short LPS (Figure 2; Tables 1 and S3). Thus, changes in lifespan are likely triggered by different sets of sensory neurons in response to a variety of food-derived cues, and loss of *nmur-1* interferes with the detection of several of these cues.

The LPS dependence of the *nmur-1* phenotype makes it conceivable that *nmur-1* may regulate stress-related and innate immune responses elicited by different food sources. We find that *nmur-1* can still affect lifespan in the absence of either of three genes, *daf-16*, *hsf-1*, and *pmk-1*, all of which have major roles in stress responses and innate immunity [47]–[51],[61]–[63]. However, the mutations in *daf-16* and *hsf-1* can partly suppress the *nmur-1* lifespan phenotype (Figure 6; Table 1), which makes it possible that the *nmur-1* influence on lifespan requires a combination of mechanisms that involve *daf-16*, *hsf-1*, and/or other factors.

### Food-Type Effects on Lifespan Can Be Distinct from Food-Level Restriction

We find that the food-source influence on wild-type lifespan is strongly correlated with reproductive effects (Figure 5C and 5D), in that increases in lifespan are accompanied not only by a decreased number of total progeny but also a faster rate of reproduction. One possible interpretation of these data is that the different reproductive profiles cause the food source-dependent differences in wild-type lifespan. Indeed, with the exception of BL21, the bacterial diets we have tested seem to affect initial survival more than late-age survival. This is supported by age-specific force of mortality plots (Figure S5A): the different food sources alter wild-type mortality primarily before day 10 of adulthood but have little effect thereafter. It is conceivable that damage inflicted on somatic tissues [64] or neglect of somatic maintenance and repair during reproduction [65] are important determinants of early mortality. In agreement with this idea, we find that long-lived *glp-1* mutant worms [66], which are sterile because they generate few or no germ cells [67], have very similar lifespan, at least on OP50, HT115, CS180, and CS2429 (W. M., unpublished data). This suggests that the food type-dependent effects on wild-type lifespan are indeed germline-dependent.

Interestingly, a recent study [68] has shown that different *E. coli* food sources can differentially affect fat storage in *C. elegans*. Wild-type worms grown on HB101 or HT115 are found to have lower triacylglyceride (TAG) levels than wild-type worms grown on OP50 [68]. Although that same study and another report [68],[69] question the reliability of fat stains with vital dyes, we also observe a slightly reduced fat storage in wild type on HT115 compared to wild type on OP50, using lipid labeling with a lipophilic fluorophore (Table S4). Thus, a correlation may exist not only between lifespan and reproduction but also between lifespan and TAG levels of wild-type worms. Since germline signals have been proposed to regulate both lifespan and intestinal fat storage [70], the food-type and reproduction-dependent effects on wild-type lifespan may also be mediated by changes in TAG levels.

In contrast, we find that *nmur-1* exerts an additional effect on lifespan that is largely independent of reproduction (Figure 5C and 5D) and also appears to be independent of *glp-1* on OP50 and CS2429 (B. A. and W. M., unpublished data) and fat storage on OP50 and HT115 (Table S4). Accordingly, the *nmur-1* mutation can affect mortality prior to day 10 of adulthood (OP50 and CS180; Figure S5B) on the food sources that significantly reduce the total progeny of *nmur-1* mutants (compare OP50 and CS180 in Figures 5C, S2E, and S3C). At the same time, *nmur-1* mutants show reduced mortality after day 10, but not past day 16, of adulthood on the short LPS strains OP50 and CS2429 (Figure S5B), the latter of which has no effect on the *nmur-1* mutant number of progeny (Figure S3C). Thus, our findings imply that food sources affect lifespan through both reproduction-dependent and reproduction-independent mechanisms, with the second being uncovered by the *nmur-1* mutation.

Unlike the longevity-promoting effect of food-level restriction [5],[41], the food type-dependent effects on lifespan that we observe not only have reproduction-independent and fat storage-independent components (Figure 5C and 5D; Table S4) but are also independent of alterations in feeding rate and developmental rate (Figure 5A and 5B). In addition, our data show that different food types and *nmur-1* affect initial mortality without decreasing late-age mortality (Figure S5), again unlike food-level restriction, which decreases the slope of the mortality trajectory and thus slows the rate of aging [71]. These data lead us to propose that these two forms of dietary influence on lifespan employ distinct, but possibly overlapping, mechanisms.

Another recent study [72] has shown that different DR regimens for *C. elegans* require different signaling pathways to affect lifespan. However, some of these regimens altered not only food levels but also the nature of food sources. In fact, at least one of these protocols, which lowered protein levels, does not decrease but increase reproduction ([72] and references therein), which suggests that the lifespan effect of protein restriction, unlike that of other DR protocols, could be partly reproduction-independent. Our data might help explain some of these findings, if one assumes that the net consequence on lifespan of some DR protocols represents a mix of independent effects from food-level restriction and food-type dependence. In the future, it would be of interest to determine whether the food type-dependent effects on lifespan will also require the activities of genes, e.g., the NFE2-related protein *skn-1* [38] and the FOXA transcription factor *pha-4* [73], that have been implicated in the longevity-promoting effects of DR.

## Materials and Methods

### Worm Strains and Bacterial Strains

All worm mutant strains used in this study were backcrossed six times to our lab wild-type (N2) strain, with the exception of *nmur-1(ok1387)*, which was backcrossed eight times, and *eat-2(ad1116)*, which was outcrossed once, before generation of different mutant combinations and any phenotypic analysis. The different worm mutant alleles used are indicated within the figures, supplementary tables, and their legends. Worms were grown for at least two generations at 25°C on the same food source used in a given phenotypic analysis, unless otherwise stated.

The *E. coli* strains used were: OP50 [11], HT115 [rnc14::ΔTn10 λ(DE3) of W3110] [13],[26],[27], BL21(DE3) [28], DY330(DE3) [Δ(argF-lacZ)U169 gal490*(IS2) pglΔ8 rnc<>cat λcI857 Δ(cro-bioA) of W3110] [30], HB101 [29], DH5α [31], CS180 [rfa+] [35], CS2198 [rfaJ19::Tnlac Δlac pyrD+ of CS180] [35], CS2429 [rfaC^−^ of CS180] [36], and CS1861 (CS180 transformed with a plasmid that confers chloramphenicol resistance and encodes the proteins required for the expression of *Shigella dysenteriae* 1 O Antigen fused to the parent strain K-12 LPS) [36].

### Transgenic Worms

We generated two independent rescue lines using standard methods: *nmur-1(ok1387)*; *jxEx12[nmur-1p::nmur-1+myo-3p::rfp]* and *nmur-1(ok1387)*; *jxEx40[nmur-1p::nmur-1+myo-3p::rfp]*. The rescue fragment, which is a 7.96 kb-long PCR fragment of the wild-type *nmur-1* genomic locus (injected at 100 ng/µl), includes the 2.9 kb sequence upstream of the *nmur-1* start codon and the 1 kb sequence downstream of the correct stop codon (see Figure S1). The *myo-3p::rfp* (gift of Cori Bargmann) was used as a coinjection marker (injected at 100 ng/µl). As controls, we also generated wild-type and *nmur-1* mutant worms that carry the *myo-3p::rfp* coinjection marker alone.

We observed that the extrachromosomal array *jxEx12* has a large number of arrested embryos and larvae, whereas the extrachromosomal array *jxEx40* produces ∼13% arrested larvae (25 arrested worms/196 total worms). These additional phenotypes might be due to a hyperactive NMUR-1 pathway caused by overexpression of the gene from its extrachromosomal copies.

To determine the expression pattern of *nmur-1*, we generated a transcriptional *gfp* reporter construct (*nmur-1p::gfp*; based on the pPD117.01 vector; gift from A. Fire), in which the *gfp* is flanked by the 2.9 kb sequence upstream of the *nmur-1* start codon and by the 1 kb sequence downstream of the correct stop codon, including the newly identified 3′ UTR (see Figure S1). In addition, sequences from the four largest introns, 1, 4, 8, and 10, which may contain regulatory sequences required for expression, were fused downstream of the 1 kb 3′ *cis* sequences. This construct was injected into wild-type worms at a concentration of 100 ng/µl, and two independent transgenic lines, *jxEx36* and *jxEx37*, were recovered, which show identical patterns of *gfp* expression.

### Bacterial Culture and Assay Plate Preparation

All bacterial strains were grown from single colonies in Luria-Bertani medium overnight at 37°C. However, the medium used to grow the chloramphenicol-resistant strain CS1861 was supplemented with 100 µg/ml chloramphenicol. Nematode-growth agar plates (6 cm in diameter; [11]) were seeded with 100 µl bacterial culture and were allowed to dry at room temperature (23°C). Seeded plates were stored at room temperature and used within 5 d.

### Lifespan Assays

The survival analyses of all worm strains on the different bacteria were initiated on the first day of adulthood and carried out at 25°C. Throughout their reproductive period, the worms were transferred daily to new plates to separate them from their progeny. We used the JMP 5.1 (SAS) software to determine Kaplan-Meier estimates of survival probabilities and mean lifespan, and for all statistical comparisons. *p* values were determined by the logrank and Wilcoxon tests. The logrank test, which places more weight on larger survival times, is appropriate when comparing differences between groups of animals whose ratio of hazard functions (ratio of mortality rates) stays approximately constant over time [74]. However, when the hazard ratios do not stay constant with time, as when one survival curve shows more early deaths than another (e.g., wild type on OP50 versus wild type on HB101 or HT115 in Figure 1A and 1B), the Wilcoxon test is more appropriate for comparing differences between groups [74]. We found that the Wilcoxon test is more sensitive to the lifespan differences we see in most of our experiments, since the *nmur-1* mutation and most bacterial food sources clearly affect mean lifespan more than the maximum lifespan, which in fact violates the logrank test assumption of constant hazard ratios. Here we refer to a Wilcoxon *p* value of ≤0.01 as a significant difference between the various groups of animals. For comparison, we report both the Wilcoxon and logrank test results in all tables.

For mortality plots, the age-specific force of mortality was calculated as F_x_ = −ln(1−D_x_), where D_x_ is the probability of death between day x−1 and x of adulthood [75]. At least five independent trials of a given lifespan experiment were used to calculate means and standard errors of F_x_, which were plotted on a log scale against age.

### Measurements of Feeding Rate, Progeny Number, and Rate of Development

Feeding rates were determined on the first and fourth days of adulthood at 25°C by measuring the animals' pharyngeal pumping rates, which reflect the rates at which they eat bacteria [76]. The pumps of the pharyngeal bulbs of individual worms were counted 3 to 5 times over periods of 30 s. Each resulting mean value was then doubled to get “pumps per minute.” A two-way ANOVA test was used to compare the different genotypes on different food sources and *p* values were calculated with the Tukey post-test.

Developmental rate differences were determined through a population-based assay at 25°C. First-stage (L1) larvae that had hatched within a 2-h time window were collected and allowed to develop for 36.5 h. At this point, the number of second-stage (L2), third-stage (L3), and fourth-stage (L4) larvae, as well as of young adult (YA) or gravid adult (GA) worms were counted. The chi-square test was used to compare the resulting stage distributions across food sources or worm genotypes.

Total progeny and temporal profiles of egg-laying were determined at 25°C by culturing L4 larvae singly on plates of the appropriate food source. The worms were then transferred to new plates regularly until they stopped laying eggs. The eggs were allowed to hatch and the larval progeny were then counted. Two-way ANOVA and the Tukey post-test were used to compare the total number of progeny of genotypes across food sources. To ensure that the data followed a normal distribution, it was necessary to incorporate a statistical censoring procedure to exclude outliers (worms with a very low number of progeny) from the data set before the ANOVA test. Briefly, this involved the tentative identification of outliers and calculation of standard deviation (SD) for the remaining set. Then, from the full data set, we excluded worms that had produced less progeny than the mean minus 2.5 times SD. In general, this procedure led to exclusion of worms with a progeny number smaller than 90, which corresponded to ∼4% of the total data set. The exception is *nmur-1* mutant worms feeding on HB101, for which two classes of worms seem to exist: one with a large number of progeny and another with a small number of progeny. In this particular case, censoring caused 25% of worms to be excluded from the analysis and the remaining data set to be biased considerably towards a larger progeny number, as can be seen in Figure S2E.

The temporal profiles of egg-laying were determined from the same statistically censored populations of worms. The Hill function, P(t) = P_max_ * t^n^/(t^n^+t_50_^n^), was used to fit the cumulative number of progeny over time, where t denotes time, P_max_ is the total number of progeny, n the Hill coefficient, and t_50_ the time until half of the progeny is produced. In Figures S2F and S3D, the data were normalized to P_max_.

For statistical assessments of correlations between mean lifespan and feeding, development, or reproduction on different food sources, we used the Pearson Product Moment test. To determine the correlation between lifespan and development, the stage distributions of the original data were used to calculate “speed of development” values, which are the percentages of worms scored as either young or gravid adults in the corresponding assays. *eat-2* mutants have a value of zero on this scale because no mutant worms reached adulthood within 36.5 h after egg-laying. To correlate lifespan and rate of reproduction, the t_50_ of the fitted temporal reproduction profiles was used.

## Supporting Information

Figure S1 **Gene architecture and coding sequence of** ***nmur-1*** **.** (A) The gene structure of *nmur-1* (*C48C5.1*) predicted by WormBase (version WS207; www.wormbase.org) consists of only 10 exons (shown in white). However, upon isolation and sequencing of the *nmur-1* cDNA, we found that the *nmur-1* gene locus includes a terminal 11th exon (shown in black) that encodes an additional 22 amino acids and is followed by a 210 bp 3′ UTR (gray). The extent of the *ok1387* deletion is indicated by the hatched bar. (B) The *nmur-1* cDNA sequence along with its translated protein sequence. The arrowheads indicate exon-intron boundaries within the DNA sequence, the 3′ UTR is italicized and the poly-A sequence used for priming the reverse transcription of the mRNA is framed. Within the protein sequence, the predicted seven transmembrane domains are underlined. The revised protein sequence shows 45% similarity and 27% identity to human NMUR1 and 47% similarity and 29% identity to human NMUR2 [24].
(0.37 MB TIF)

Figure S2 ***nmur-1*** **modulates food source-dependent effects on feeding rate, development, and reproduction.** (A–B) Pharyngeal pumping rates of wild-type and mutant worms on different bacteria. Rates are expressed as mean pumps per minute and determined from the indicated number (n) of worms. ** indicates *p*≤0.001 in this and subsequent panels. Since wild type and *nmur-1* mutants pump at a similar rate on HB101, a food source that does increase mutant lifespan compared to wild type (Figure 2E), the *nmur-1* regulation of lifespan and feeding rate presumably involve two distinct pathways. (C) The wild-type *nmur-1* genomic locus can also rescue the feeding rate phenotypes of *nmur-1* mutants on OP50 (*p* = 0.02) and HT115 (*p*≤0.001). The rescued worms are compared to wild-type and *nmur-1* mutant worms that carry the *myo-3p::rfp* coinjection marker alone. * indicates *p*≤0.01 in this and later panels. (D) Distribution of developmental stages of wild-type and mutant worms at 36.5 h after hatching on different bacteria. L2, second-stage larvae; L3, third-stage larvae; L4, fourth-stage larvae; YA, young adults. Although both wild type and mutants develop faster on OP50 than on HB101 or HT115 (*p*<0.001 for either genotype), mutants develop slower than wild type on OP50 (*p* = 0.01). It should be noted that our observation of a slower wild-type developmental rate on HB101 at 25°C differs from a previous study carried out at 18°C [77], which suggests that temperature can alter the growth-influencing factors of some food sources. (E–F) Total progeny and temporal profiles of reproduction on different bacteria. *nmur-1* mutants have less total progeny (E) than wild type on OP50 (*p*<0.01), and wild type has more progeny on OP50 than on HT115 (*p* = 0.03). The larger progeny number of *nmur-1* mutants on HB101 (*p* = 0.04) is a consequence of censoring (see Materials and Methods). *nmur-1* mutants reproduce more slowly (F) than wild type on HT115 but behave more similarly to wild type on OP50 and HB101.
(0.88 MB TIF)

Figure S3 **The influence of the LPS structure on feeding, development, and reproduction of wild-type and** ***nmur-1*** **mutant worms.** (A) Wild-type and mutant worms have similar pharyngeal pumping rates on both the CS2429 LPS truncation mutant and the CS180 parent strain. (B) *nmur-1* mutant worms develop faster on the *E. coli* LPS mutant strain than on the *E. coli* parent strain (*p*<0.001) but slower than wild-type worms on both *E. coli* strains (*p*<0.001 for each case). *nmur-1* mutants also (C) produce more offspring on the *E. coli* truncation mutant than on the *E. coli* parent strain (** *p*<0.001) and (D) reproduce at a similar rate, though slower than wild type, on both strains. Together our findings suggest that the *nmur-1* regulation of lifespan, feeding rate, development, and reproduction involve more than one pathway and several food-derived factors.
(0.28 MB TIF)

Figure S4 **The effect of a genetic model of food-level restriction on feeding, reproduction, and lifespan.** Worms carrying the mutation *eat-2(ad1116)* display a reduced pharyngeal pumping rate (A), a smaller number of progeny (B), and increased lifespan (C) independent of their food source. Mean lifespan of *eat-2* mutants: 16.8 d (+47%, *p*<0.0001) on OP50, 15.9 d (+41%, *p*<0.0001) on HT115.
(0.39 MB TIF)

Figure S5 **Food source-dependent effects on age-specific rates of mortality.** (A) Mortality plot of wild type on four different strains of *E. coli*. (B) Individual comparisons of wild-type and *nmur-1* mutants on the four food sources.
(0.68 MB TIF)

Table S1 **Sensory neurons affected by cilium-structure genes.** The subsets of sensory neurons affected by two sensory genes, *daf-10* [15] and *osm-3* [16], partly overlap. The superscripted symbols indicate the references that identify the neurons and their corresponding functions: ^a^, [6]; ^b^, [78],[79],[80]; ^c^, [81]; ^d^, [82]; ^e^, [52]; and ^f^, [83].
(0.03 MB DOC)

Table S2 **Adult lifespans of neuropeptide and neuropeptide receptor mutants tested at 25°C.** We measured the lifespan of *C. elegans* grown on OP50 or HT115 and that carry mutations in genes that encode either neuropeptides or neuropeptide receptors. These neuropeptides or neuropeptide receptors show homologies to members of different neuropeptide signaling pathways in other animals, which are involved in regulating their feeding behavior and metabolism [19],[21]–[25]. The statistical analyses performed on these experiments are as described in the legend of Table 1.
(0.08 MB DOC)

Table S3 **Individual trials of adult lifespans on different food sources at 25°C.** The analyses performed here are as described in the legend of Table 1. The superscripted symbols indicate the following: ^a^, compared to the same genotype assayed in parallel on OP50 in independent trials; ^b^, compared to *jxEx4[myo-3p::rfp]* on the same food source in independent trials; ^c^, compared to the rescue line on the same food source in independent trials; and ^d^, compared to the same genotype assayed in parallel on CS180 in independent trials.
(0.27 MB DOC)

Table S4 **Fat storage of wild-type and** ***nmur-1*** **mutant worms on OP50 and HT115.** Fat storage in 1-d-old adults is quantified by labeling the worms with C1-BODIPY-C12 according to Mak et al. [84]. All quantifications are normalized to wild type on OP50 and given as percent ± SEM. Numbers in parentheses indicate the number of worms assayed for each condition. The superscript ^a^ indicates *p* = 0.042 compared to wild type on OP50.
(0.03 MB DOC)

## Abbreviations

DR: dietary restriction
LPS: lipopolysaccharide
NMU: neuromedin U
NMUR: neuromedin U receptor
TAG: triacylglyceride
