# Supplementary figures and images for: A Neuromedin U Receptor Acts with the Sensory System to Modulate Food Type-Dependent Effects on C. elegans Lifespan
Source: PLoS Biol. 2010 May 25;8(5):e1000376. doi: 10.1371/journal.pbio.1000376 (PMC2876044; doi:10.1371/journal.pbio.1000376)

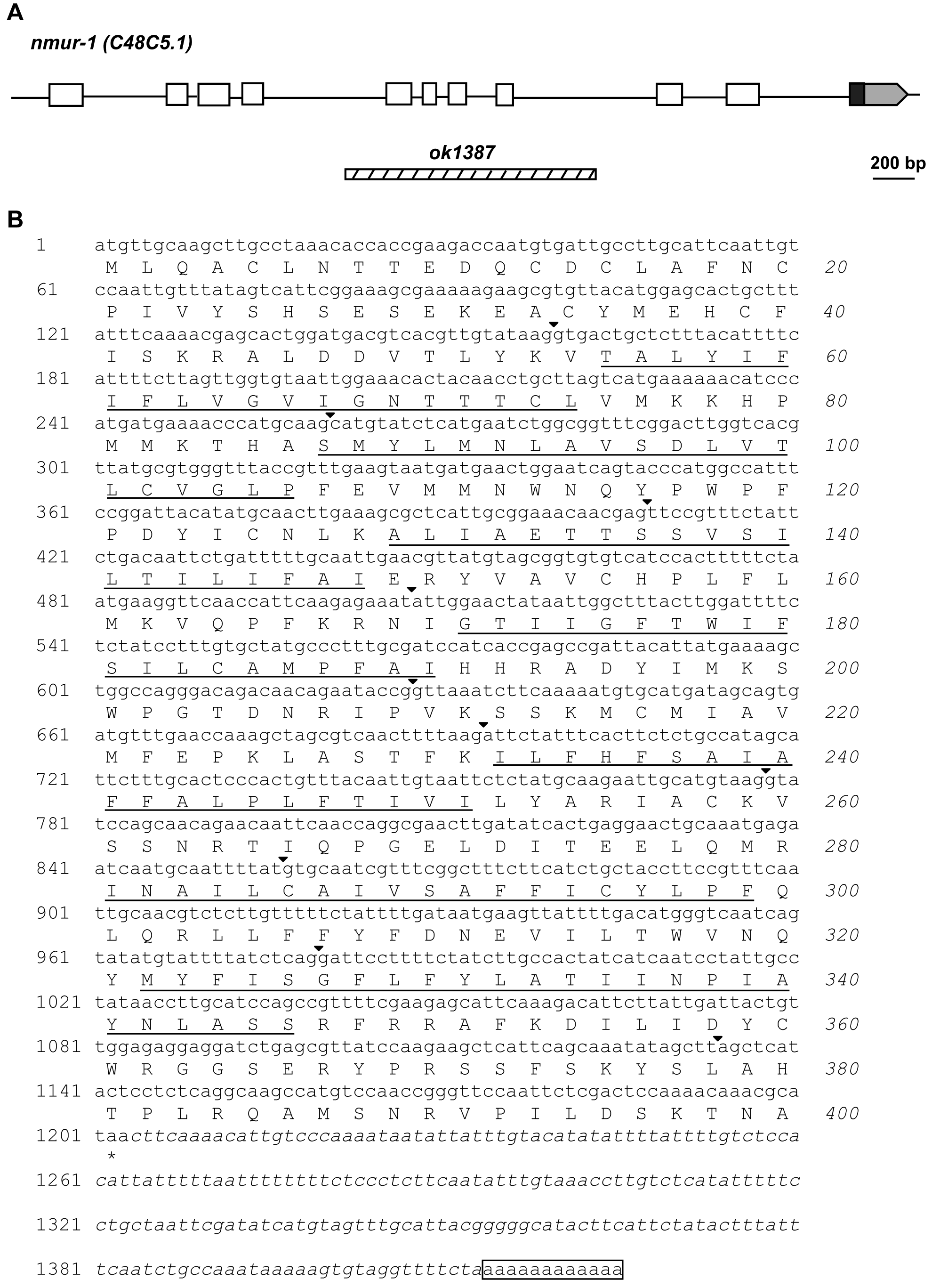

Supplement: Figure S1 — Gene architecture and coding sequence of nmur-1 . (A) The gene structure of nmur-1 (C48C5.1) predicted by WormBase (version WS207; www.wormbase.org) consists of only 10 exons (shown in white). However, upon isolation and sequencing of the nmur-1 cDNA, we found that the nmur-1 gene locus includes a terminal 11th exon (shown in black) that encodes an additional 22 amino acids and is followed by a 210 bp 3′ UTR (gray). The extent of the ok1387 deletion is indicated by the hatched bar. (B) The nmur-1 cDNA sequence along with its translated protein sequence. The arrowheads indicate exon-intron boundaries within the DNA sequence, the 3′ UTR is italicized and the poly-A sequence used for priming the reverse transcription of the mRNA is framed. Within the protein sequence, the predicted seven transmembrane domains are underlined. The revised protein sequence shows 45% similarity and 27% identity to human NMUR1 and 47% similarity and 29% identity to human NMUR2 [24]. (0.37 MB TIF) [file pbio.1000376.s001.tif]

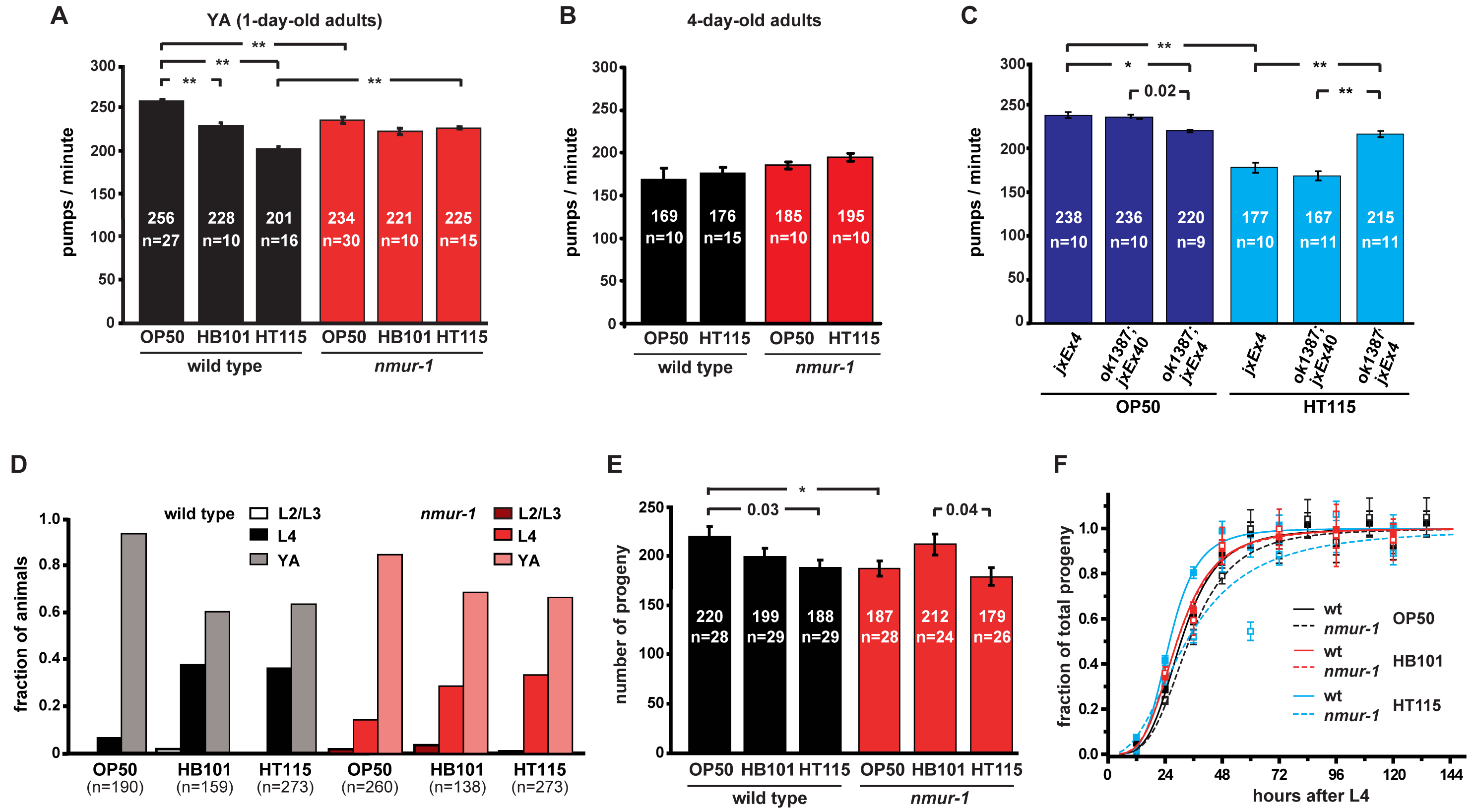

Supplement: Figure S2 — nmur-1 modulates food source-dependent effects on feeding rate, development, and reproduction. (A–B) Pharyngeal pumping rates of wild-type and mutant worms on different bacteria. Rates are expressed as mean pumps per minute and determined from the indicated number (n) of worms. ** indicates p≤0.001 in this and subsequent panels. Since wild type and nmur-1 mutants pump at a similar rate on HB101, a food source that does increase mutant lifespan compared to wild type (Figure 2E), the nmur-1 regulation of lifespan and feeding rate presumably involve two distinct pathways. (C) The wild-type nmur-1 genomic locus can also rescue the feeding rate phenotypes of nmur-1 mutants on OP50 (p = 0.02) and HT115 (p≤0.001). The rescued worms are compared to wild-type and nmur-1 mutant worms that carry the myo-3p::rfp coinjection marker alone. * indicates p≤0.01 in this and later panels. (D) Distribution of developmental stages of wild-type and mutant worms at 36.5 h after hatching on different bacteria. L2, second-stage larvae; L3, third-stage larvae; L4, fourth-stage larvae; YA, young adults. Although both wild type and mutants develop faster on OP50 than on HB101 or HT115 (p<0.001 for either genotype), mutants develop slower than wild type on OP50 (p = 0.01). It should be noted that our observation of a slower wild-type developmental rate on HB101 at 25°C differs from a previous study carried out at 18°C [77], which suggests that temperature can alter the growth-influencing factors of some food sources. (E–F) Total progeny and temporal profiles of reproduction on different bacteria. nmur-1 mutants have less total progeny (E) than wild type on OP50 (p<0.01), and wild type has more progeny on OP50 than on HT115 (p = 0.03). The larger progeny number of nmur-1 mutants on HB101 (p = 0.04) is a consequence of censoring (see Materials and Methods). nmur-1 mutants reproduce more slowly (F) than wild type on HT115 but behave more similarly to wild type on OP50 and HB101. (0.88 MB TIF) [file pbio.1000376.s002.tif]

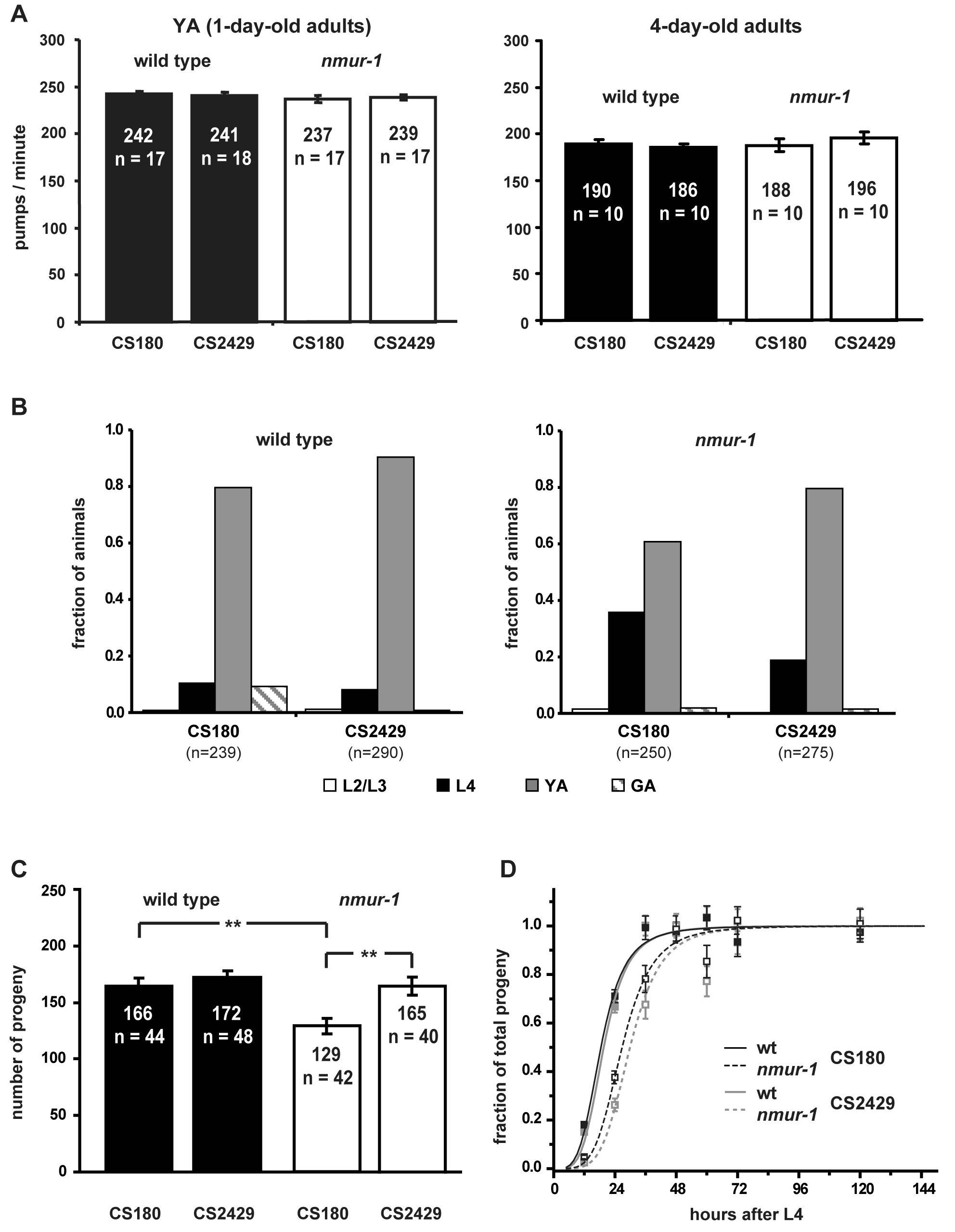

Supplement: Figure S3 — The influence of the LPS structure on feeding, development, and reproduction of wild-type and nmur-1 mutant worms. (A) Wild-type and mutant worms have similar pharyngeal pumping rates on both the CS2429 LPS truncation mutant and the CS180 parent strain. (B) nmur-1 mutant worms develop faster on the E. coli LPS mutant strain than on the E. coli parent strain (p<0.001) but slower than wild-type worms on both E. coli strains (p<0.001 for each case). nmur-1 mutants also (C) produce more offspring on the E. coli truncation mutant than on the E. coli parent strain (** p<0.001) and (D) reproduce at a similar rate, though slower than wild type, on both strains. Together our findings suggest that the nmur-1 regulation of lifespan, feeding rate, development, and reproduction involve more than one pathway and several food-derived factors. (0.28 MB TIF) [file pbio.1000376.s003.tif]

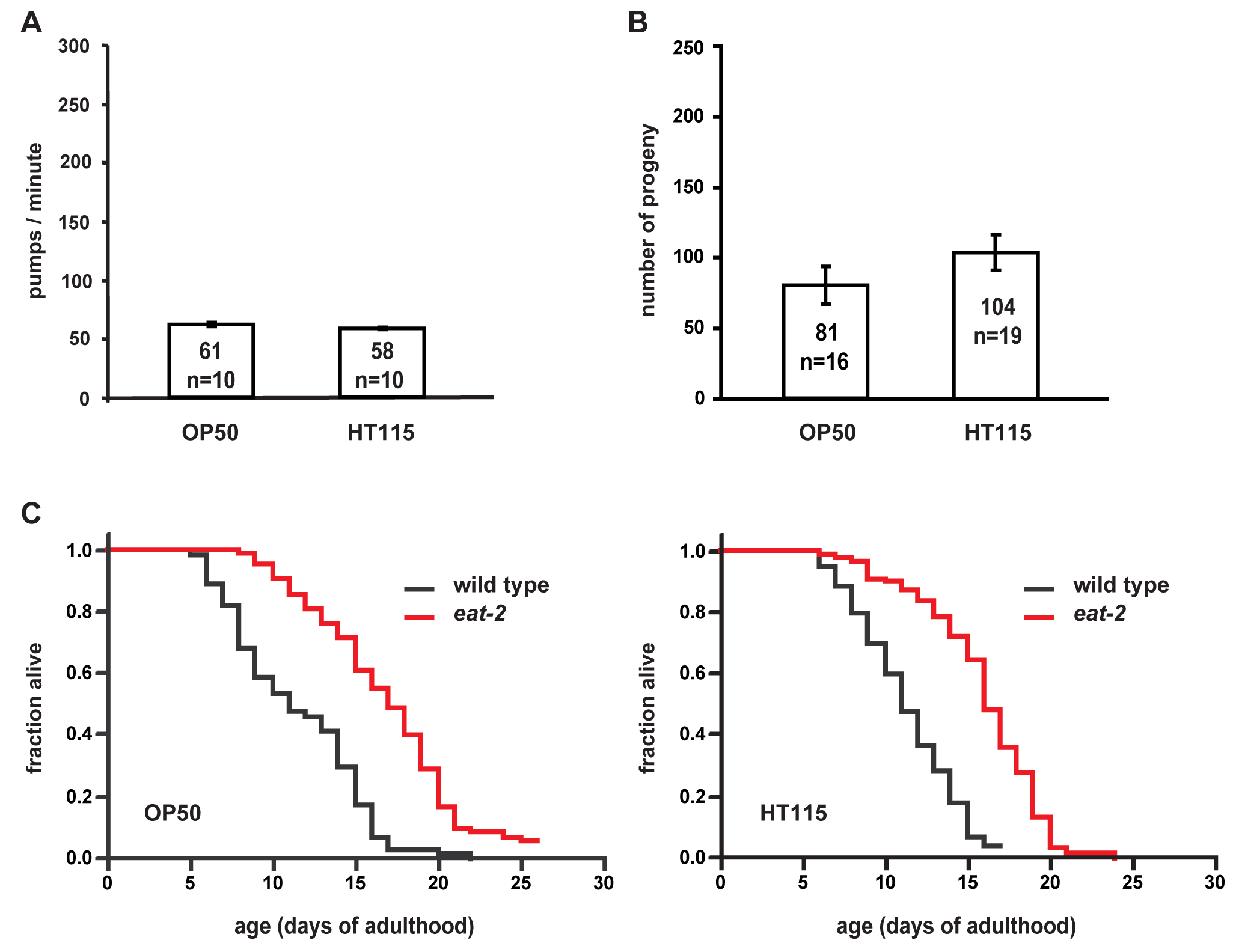

Supplement: Figure S4 — The effect of a genetic model of food-level restriction on feeding, reproduction, and lifespan. Worms carrying the mutation eat-2(ad1116) display a reduced pharyngeal pumping rate (A), a smaller number of progeny (B), and increased lifespan (C) independent of their food source. Mean lifespan of eat-2 mutants: 16.8 d (+47%, p<0.0001) on OP50, 15.9 d (+41%, p<0.0001) on HT115. (0.39 MB TIF) [file pbio.1000376.s004.tif]

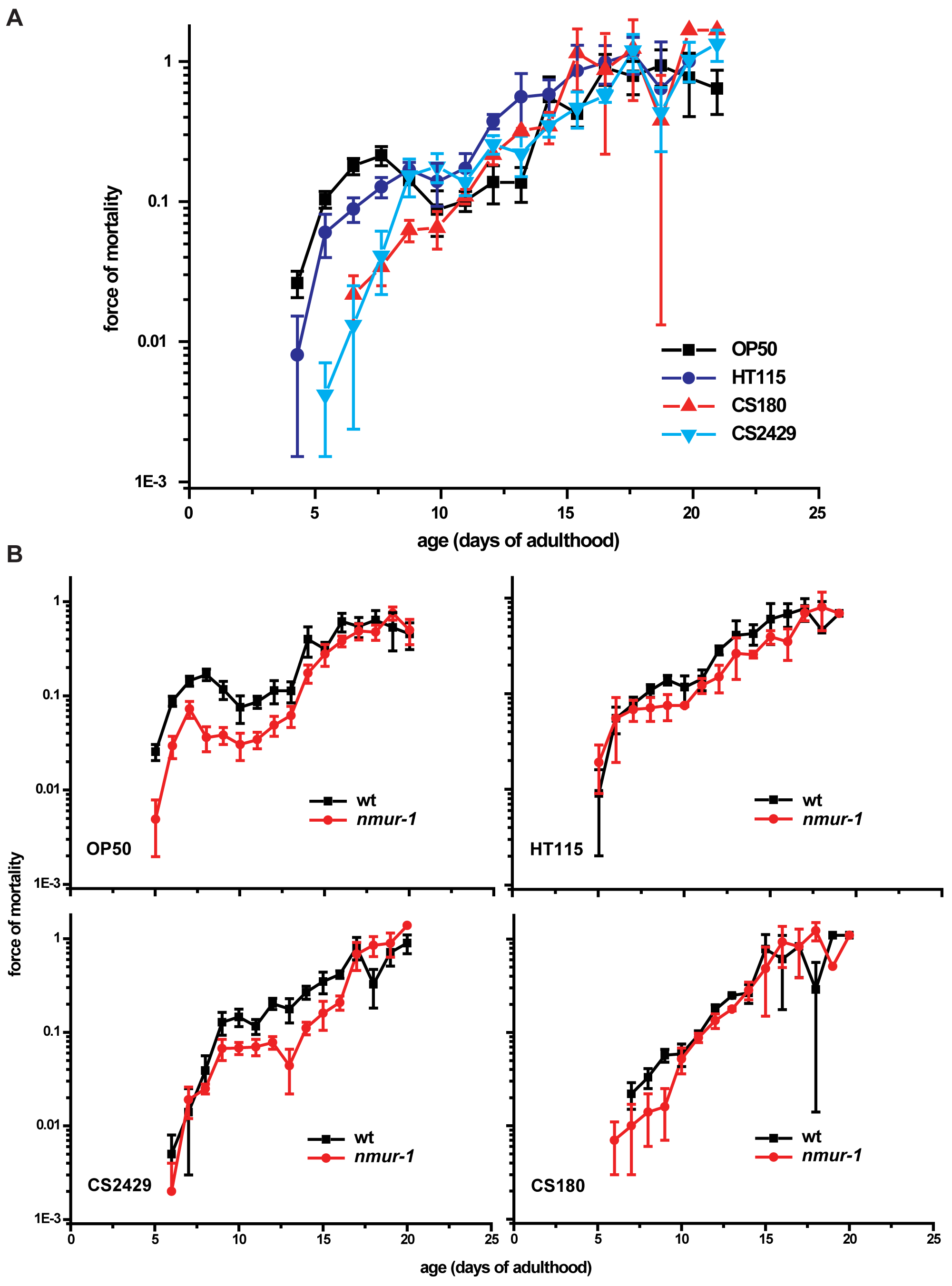

Supplement: Figure S5 — Food source-dependent effects on age-specific rates of mortality. (A) Mortality plot of wild type on four different strains of E. coli. (B) Individual comparisons of wild-type and nmur-1 mutants on the four food sources. (0.68 MB TIF) [file pbio.1000376.s005.tif]
