# Supplementary material for: A Neuromedin U Receptor Acts with the Sensory System to Modulate Food Type-Dependent Effects on C. elegans Lifespan
Source: PLoS Biol. 2010 May 25;8(5):e1000376. doi: 10.1371/journal.pbio.1000376 (PMC2876044; doi:10.1371/journal.pbio.1000376)
Supplement: Table S1 — Sensory neurons affected by cilium-structure genes. The subsets of sensory neurons affected by two sensory genes, daf-10 [15] and osm-3 [16], partly overlap. The superscripted symbols indicate the references that identify the neurons and their corresponding functions: a, [6]; b, [78],[79],[80]; c, [81]; d, [82]; e, [52]; and f, [83]. (0.03 MB DOC) [file pbio.1000376.s006.doc]

Supplementary Table 1. Sensory neurons affected by cilium-structure genes

| **Gene** | **Sensory Neurons Affected** | **Function** |
| --- | --- | --- |
| *daf-10* | Amphid sensory neurons | Chemosensation^a,b^, mechanosensation^a,c^,  Thermosensation^a,d^ |
|  | Phasmid sensory neurons | Chemosensation^a,e^ |
|  | CEP | Dopaminergic mechanosensory neuron^a,f^ |
| *osm-3* | ADF, ADL, ASE, ASG, ASH, ASI, ASJ, ASK | Amphid sensory neurons^a^; chemosensation^b^ |
|  | IL2 | Inner labial sensory neurons^a^ |
|  | PHA, PHB | Phasmid sensory neurons^a^; chemosensation^e^ |
