# Supplementary material for: A Neuromedin U Receptor Acts with the Sensory System to Modulate Food Type-Dependent Effects on C. elegans Lifespan
Source: PLoS Biol. 2010 May 25;8(5):e1000376. doi: 10.1371/journal.pbio.1000376 (PMC2876044; doi:10.1371/journal.pbio.1000376)
Supplement: Table S2 — Adult lifespans of neuropeptide and neuropeptide receptor mutants tested at 25°C. We measured the lifespan of C. elegans grown on OP50 or HT115 and that carry mutations in genes that encode either neuropeptides or neuropeptide receptors. These neuropeptides or neuropeptide receptors show homologies to members of different neuropeptide signaling pathways in other animals, which are involved in regulating their feeding behavior and metabolism [19],[21]–[25]. The statistical analyses performed on these experiments are as described in the legend of Table 1. (0.08 MB DOC) [file pbio.1000376.s007.doc]

Supplementary Table 2. Adult lifespans of neuropeptide and neuropeptide receptor mutants tested at 25^o^C

| ORF/  Treatment |  | Allele | Homolog | No. of Animals Observed/Total Initial Animals | %  Wild type | *P* Value Against Wild type  (Logrank) | *P* Value Against Wild type  (Wilcoxon) |
| --- | --- | --- | --- | --- | --- | --- | --- |
| *E. coli* OP50 |  |  |  |  |  |  |  |
| *C30F12.6* | *nmur-4* | *ok1381* | Thyrotropin-releasing hormone-like receptor/  Neuromedin U-like receptor | 57/80 | + 4 | 0.55 | 0.82 |
| *C48C5.1* | *nmur-1* | *ok1387* | Neuromedin U-like receptor | 61/70 | **+ 34** | 0.002 | 0.0003 |
| *C50H2.1* | *fshr-1* | *ok778* | Follicle stimulating hormone receptor | 63/176 | - 5 | 0.39 | 0.28 |
| *F35B12.7* | *nlp-24* | *tm2105* | Opioid-like neuropeptide | 50/68 | + 9 | 0.13 | 0.09 |
| *F35C11.1* | *nlp-5* | *tm2125* | Allatostatin/  galanin-like neuropeptide | 85/98 | + 8 | 0.05 | 0.10 |
| *F56B6.5* | *npr-16* | *ok1541* | Somatostatin-like receptor | 61/80 | + 7 | 0.28 | 0.32 |
| *H02I12.3* | *tag-89* | *ok514* | Thyrotropin-releasing hormone-like receptor | 64/80 | + 9 | 0.21 | 0.26 |
| *K10B4.4* | *nmur-2* | *ttTi8340* | Neuromedin U-like receptor | 66/80 | + 2 | 0.74 | 0.56 |
| *ZK455.3* | *npr-9* | *tm1652* | Galanin-like receptor | 67/80 | + 2 | 0.42 | 0.84 |
|  |  |  |  |  |  |  |  |
|  |  |  |  |  |  |  |  |
|  |  |  |  |  |  |  |  |
| ORF/  Treatment |  | Allele | Homolog | No. of Animals Observed/  Total Initial Animals | %  Wild type | *P* Value Against Wild type  (Logrank) | *P* Value Against Wild type  (Wilcoxon) |
| *E. coli* HT115 |  |  |  |  |  |  |  |
| *C30F12.6* | *nmur-4* | *ok1381* | Thyrotropin-releasing hormone-like receptor/  Neuromedin U-like receptor | 58/80 | - 7 | 0.56 | 0.27 |
| *C48C5.1* | *nmur-1* | *ok1387* | Neuromedin U-like receptor | 50/60 | - 6 | 0.51 | 0.32 |
| *C50H2.1* | *fshr-1* | *ok778* | Follicle stimulating hormone receptor | 36/70 | - 8 | 0.04 | 0.06 |
| *F35B12.7* | *nlp-24* | *tm2105* | Opioid-like neuropeptide | 58/70 | + 5 | 0.54 | 0.43 |
| *F35C11.1* | *nlp-5* | *tm2125* | Allatostatin/  galanin-like neuropeptide | 60/70 | + 3 | 0.31 | 0.61 |
| *F56B6.5* | *npr-16* | *ok1541* | Somatostatin-like receptor | 65/71 | + 1 | 0.84 | 0.94 |
| *H02I12.3* | *tag-89* | *ok514* | Thyrotropin-releasing hormone-like receptor | 64/70 | + 6 | 0.32 | 0.28 |
| *K10B4.4* | *nmur-2* | *ttTi8340* | Neuromedin U-like receptor | 51/79 | - 8 | 0.10 | 0.30 |
| *ZK455.3* | *npr-9* | *tm1652* | Galanin-like receptor | 60/70 | + 8 | 0.06 | 0.13 |
|  |  |  |  |  |  |  |  |
