# Supplementary material for: A Neuromedin U Receptor Acts with the Sensory System to Modulate Food Type-Dependent Effects on C. elegans Lifespan
Source: PLoS Biol. 2010 May 25;8(5):e1000376. doi: 10.1371/journal.pbio.1000376 (PMC2876044; doi:10.1371/journal.pbio.1000376)
Supplement: Table S3 — Individual trials of adult lifespans on different food sources at 25°C. The analyses performed here are as described in the legend of Table 1. The superscripted symbols indicate the following: a, compared to the same genotype assayed in parallel on OP50 in independent trials; b, compared to jxEx4[myo-3p::rfp] on the same food source in independent trials; c, compared to the rescue line on the same food source in independent trials; and d, compared to the same genotype assayed in parallel on CS180 in independent trials. (0.27 MB DOC) [file pbio.1000376.s008.doc]

*Supplementary Table 3. Individual trials of adult lifespans on different food sources at 25^o^C*

| Strain/Treatment | Mean Lifespan  ± SEM (Days) | 75^th^ Percentile (Days) | No. of Animals Observed/  Total Initial Animals | %  Wild type | *P* Value Against Wild type  (Logrank) | *P* Value Against Wild type  (Wilcoxon) | % of Specified Groups | *P* Value Against Specified Groups  (Logrank) | *P* Value Against Specified Groups  (Wilcoxon) |
| --- | --- | --- | --- | --- | --- | --- | --- | --- | --- |
| Sensory mutants |  |  |  |  |  |  |  |  |  |
| Trial 1 - OP50: Wild type | 11.7 ± 0.5 | 15 | 71/80 |  |  |  |  |  |  |
| Trial 1 - OP50: *daf-10(m79)* | 15.8 ± 1.3 | 27 | 39/78 | *+ 35* | 0.004 | 0.15 |  |  |  |
| Trial 1 - HT115: Wild type | 12.2 ± 0.4 | 15 | 68/70 |  |  |  | + 4 | 0.79^a^ | 0.17^a^ |
| Trial 1 - HT115: *daf-10(m79)* | 17.3 ± 1.1 | 24 | 56/70 | **+ 42** | < 0.0001 | 0.004 | + 9 | 0.29^a^ | 0.15^a^ |
| Trial 2 - OP50: Wild type | 10.9 ± 0.5 | 15 | 59/70 |  |  |  |  |  |  |
| Trial 2 - OP50: *daf-10(m79)* | 17.6 ± 1.2 | 24 | 39/70 | **+ 61** | < 0.0001 | < 0.0001 |  |  |  |
| Trial 2 - HT115: Wild type | 11.9 ± 0.4 | 15 | 56/70 |  |  |  | + 9 | 0.58^a^ | 0.05^a^ |
| Trial 2 - HT115: *daf-10(m79)* | 18.2 ± 1.1 | 24 | 45/70 | **+ 53** | < 0.0001 | 0.0002 | + 3 | 0.75^a^ | 0.62^a^ |
|  |  |  |  |  |  |  |  |  |  |
| Trial 1 - OP50: Wild type | 10.9 ± 0.5 | 15 | 59/70 |  |  |  |  |  |  |
| Trial 1 - OP50: *osm-3(n1540)* | 13.7 ± 0.6 | 17 | 52/70 | **+ 26** | 0.0002 | 0.0005 |  |  |  |
| Trial 1 - HT115: Wild type | 11.9 ± 0.4 | 15 | 56/70 |  |  |  | + 9 | 0.58^a^ | 0.05^a^ |
| Trial 1 - HT115: *osm-3(n1540)* | 11.4 ± 0.6 | 15 | 55/70 | - 4 | 0.49 | 0.25 | **- 17** | 0.08^a^ | 0.01^a^ |
| Trial 2 - OP50: Wild type | 9.2 ± 0.5 | 12 | 61/70 |  |  |  |  |  |  |
| Trial 2 - OP50: *osm-3(n1540)* | 12.5 ± 0.4 | 15 | 78/100 | **+ 36** | < 0.0001 | < 0.0001 |  |  |  |
| Trial 2 - HT115: Wild type | 12.3 ± 0.4 | 15 | 66/70 |  |  |  | **+ 34** | 0.0003^a^ | < 0.0001^a^ |
| Trial 2 - HT115: *osm-3(n1540)* | 13.2 ± 0.4 | 16 | 90/100 | + 7 | 0.03 | 0.09 | + 6 | 0.11^a^ | 0.16^a^ |
| Trial 3 - OP50: Wild type | 11.3 ± 0.5 | 15 | 67/80 |  |  |  |  |  |  |
| Trial 3 - OP50: *osm-3(n1540)* | 12.8 ± 0.6 | 16 | 62/80 | *+ 13* | 0.01 | 0.06 |  |  |  |
| Trial 3 - HT115: Wild type | 11.3 ± 0.4 | 15 | 76/80 |  |  |  | 0 | 0.50^a^ | 0.91^a^ |
| Trial 3 - HT115: *osm-3(n1540)* | 12.7 ± 0.5 | 16 | 74/80 | *+ 12* | 0.004 | 0.02 | - 1 | 0.42^a^ | 0.95^a^ |
|  |  |  |  |  |  |  |  |  |  |
| *nmur-1* food-dependence |  |  |  |  |  |  |  |  |  |
| Trial 1 - OP50: Wild type | 10.5 ± 0.5 | 14 | 73/80 |  |  |  |  |  |  |
| Trial 1 - OP50: *nmur-1* | 15.7 ± 0.5 | 18 | 65/80 | **+ 50** | < 0.0001 | < 0.0001 |  |  |  |
|  |  |  |  |  |  |  |  |  |  |
| Suppl. Table 3 (Continued) |  |  |  |  |  |  |  |  |  |
| Strain/Treatment | Mean Lifespan  ± SEM (Days) | 75^th^ Percentile (Days) | No. of Animals Observed/  Total Initial Animals | %  Wild type | *P* Value Against Wild type (Logrank) | *P* Value Against Wild type  (Wilcoxon) | % of Specified Groups | *P* Value Against Specified Groups  (Logrank) | *P* Value Against Specified Groups  (Wilcoxon) |
| Trial 1 - BL21: Wild type | 7.4 ± 0.6 | 7 | 20/80 |  |  |  | **- 30** | 0.001^a^ | 0.003^a^ |
| Trial 1 - BL21: *nmur-1* | 9.9 ± 0.6 | 13 | 44/80 | **+ 34** | 0.007 | 0.004 | **- 37** | < 0.0001 ^a^ | < 0.0001^a^ |
| Trial 1 - HB101: Wild type | 13.2 ± 0.5 | 16 | 74/80 |  |  |  | **+ 26** | 0.008^a^ | < 0.0001^a^ |
| Trial 1 - HB101: *nmur-1* | 16.4 ± 0.5 | 19 | 70/80 | **+ 24** | < 0.0001 | < 0.0001 | + 4 | 0.17^a^ | 0.37^a^ |
| Trial 1 - HT115: Wild type | 12.4 ± 0.4 | 15 | 70/80 |  |  |  | **+ 18** | 0.12^a^ | 0.0007^a^ |
| Trial 1 - HT115: *nmur-1* | 13.9 ± 0.5 | 17 | 66/80 | + 12 | 0.04 | 0.02 | **- 11** | 0.001^a^ | 0.002^a^ |
| Trial 1 - DY330: Wild type | 11.9 ± 0.4 | 14 | 73/80 |  |  |  | **+ 13** | 0.28^a^ | 0.001^a^ |
| Trial 1 - DY330: *nmur-1* | 13.0 ± 0.4 | 15 | 72/80 | + 9 | 0.05 | 0.08 | **- 17** | < 0.0001^a^ | < 0.0001^a^ |
| Trial 1 - DH5α: Wild type | 12.2 ± 0.4 | 15 | 69/80 |  |  |  | **+ 16** | 0.26^a^ | 0.001^a^ |
| Trial 1 - DH5α: *nmur-1* | 13.6 ± 0.4 | 16 | 74/80 | + 11 | 0.04 | 0.02 | **- 13** | < 0.0001^a^ | < 0.0001^a^ |
|  |  |  |  |  |  |  |  |  |  |
| Trial 2 - OP50: Wild type | 10.1 ± 0.6 | 14 | 48/70 |  |  |  |  |  |  |
| Trial 2 - OP50: *nmur-1* | 13.8 ± 0.5 | 16 | 52/72 | **+ 37** | < 0.0001 | < 0.0001 |  |  |  |
| Trial 2 - BL21: Wild type | 8.1 ± 0.4 | 10 | 32/70 |  |  |  | *- 20* | 0.001^a^ | 0.05^a^ |
| Trial 2 - BL21: *nmur-1* | 10.4 ± 0.7 | 15 | 41/70 | *+ 28* | 0.008 | 0.09 | **- 25** | 0.002^a^ | 0.0002^a^ |
| Trial 2 - HB101: Wild type | 11.5 ± 0.4 | 14 | 57/70 |  |  |  | + 14 | 0.48^a^ | 0.10^a^ |
| Trial 2 - HB101: *nmur-1* | 13.9 ± 0.3 | 15 | 55/70 | **+ 21** | < 0.0001 | < 0.0001 | + 1 | 0.25^a^ | 0.62^a^ |
| Trial 2 - HT115: Wild type | 11.9 ± 0.3 | 14 | 57/70 |  |  |  | + 18 | 0.51^a^ | 0.02^a^ |
| Trial 2 - HT115: *nmur-1* | 12.6 ± 0.4 | 15 | 50/70 | *+ 6* | 0.003 | 0.08 | - 9 | 0.03^a^ | 0.05^a^ |
| Trial 2 - DY330: Wild type | 12.4 ± 0.4 | 14 | 64/70 |  |  |  | **+ 23** | 0.02^a^ | 0.005^a^ |
| Trial 2 - DY330: *nmur-1* | 12.5 ± 0.4 | 15 | 54/70 | + 1 | 0.72 | 0.64 | *- 9* | 0.01^a^ | 0.03^a^ |
| Trial 2 - DH5α: Wild type | 10.3 ± 0.2 | 12 | 62/70 |  |  |  | + 2 | 0.07^a^ | 0.72^a^ |
| Trial 2 - DH5α: *nmur-1* | 13.1 ± 0.4 | 15 | 62/70 | **+ 27** | < 0.0001 | < 0.0001 | - 5 | 0.13^a^ | 0.11^a^ |
|  |  |  |  |  |  |  |  |  |  |
| Trial 3 - OP50: Wild type | 10.9 ± 0.6 | 16 | 57/70 |  |  |  |  |  |  |
| Trial 3 - OP50: *nmur-1* | 15.2 ± 0.5 | 18 | 52/70 | **+ 39** | < 0.0001 | < 0.0001 |  |  |  |
|  |  |  |  |  |  |  |  |  |  |
| Suppl. Table 3 (Continued) |  |  |  |  |  |  |  |  |  |
| Strain/Treatment | Mean Lifespan  ± SEM (Days) | 75^th^ Percentile (Days) | No. of Animals Observed/  Total Initial Animals | %  Wild type | *P* Value Against Wild type (Logrank) | *P* Value Against Wild type  (Wilcoxon) | % of Specified Groups | *P* Value Against Specified Groups  (Logrank) | *P* Value Against Specified Groups  (Wilcoxon) |
| Trial 3 - HB101: Wild type | 11.9 ± 0.5 | 16 | 62/70 |  |  |  | + 9 | 0.74^a^ | 0.19^a^ |
| Trial 3 - HB101: *nmur-1* | 14.7 ± 0.3 | 16 | 48/70 | **+ 24** | < 0.0001 | < 0.0001 | - 3 | 0.06^a^ | 0.07^a^ |
| Trial 3 - DH5α: Wild type | 10.4 ± 0.4 | 12 | 65/70 |  |  |  | - 5 | 0.12^a^ | 1.0^a^ |
| Trial 3 - DH5α: *nmur-1* | 12.8 ± 0.3 | 16 | 61/70 | **+ 23** | < 0.0001 | < 0.0001 | **- 16** | < 0.0001^a^ | < 0.0001^a^ |
|  |  |  |  |  |  |  |  |  |  |
| Trial 4 - OP50: Wild type | 11.3 ± 0.7 | 17 | 62/70 |  |  |  |  |  |  |
| Trial 4 - OP50: *nmur-1* | 15.1 ± 0.6 | 19 | 61/70 | **+ 34** | 0.002 | 0.0003 |  |  |  |
| Trial 4 - HT115: Wild type | 13.8 ± 0.5 | 17 | 59/70 |  |  |  | **+ 22** | 0.17 ^a^ | 0.004^a^ |
| Trial 4 - HT115: *nmur-1* | 13.0 ± 0.6 | 15 | 50/60 | - 6 | 0.51 | 0.32 | **- 14** | 0.004^a^ | 0.01^a^ |
|  |  |  |  |  |  |  |  |  |  |
| Rescue experiments |  |  |  |  |  |  |  |  |  |
| Line 2 |  |  |  |  |  |  |  |  |  |
| Trial 1 |  |  |  |  |  |  |  |  |  |
| OP50: *jxEx4* | 12.5 ± 0.6 | 16 | 45/52 |  |  |  |  |  |  |
| OP50: *nmur-1; jxEx40* | 14.1 ± 0.7 | 18 | 44/72 | *+ 13* | 0.01^b^ | 0.11^b^ |  |  |  |
| OP50: *nmur-1; jxEx4* | 17.4 ± 0.6 | 21 | 55/70 | **+ 39** | < 0.0001^b^ | < 0.0001^b^ | **+ 23** | 0.0007^c^ | 0.001^c^ |
| Trial 2 |  |  |  |  |  |  |  |  |  |
| OP50: *jxEx4* | 13.1 ± 0.6 | 17 | 64/75 |  |  |  |  |  |  |
| OP50: *nmur-1; jxEx40* | 14.9 ± 0.7 | 19 | 45/80 | + 14 | 0.03^b^ | 0.02^b^ |  |  |  |
| OP50: *nmur-1; jxEx4* | 17.4 ± 0.6 | 21 | 65/74 | **+ 33** | < 0.0001^b^ | < 0.0001^b^ | **+ 17** | 0.006^c^ | 0.006^c^ |
| HT115: *jxEx4* | 11.3 ± 0.4 | 13 | 58/72 |  |  |  |  |  |  |
| HT115: *nmur-1; jxEx40* | 13.2 ± 0.5 | 17 | 80/89 | **+ 17** | 0.001^b^ | 0.005^b^ |  |  |  |
| HT115: *nmur-1; jxEx4* | 13.3 ± 0.3 | 15 | 77/82 | **+ 18** | 0.0003^b^ | 0.0001^b^ | + 1 | 0.31^c^ | 0.84^c^ |
| Trial 3 |  |  |  |  |  |  |  |  |  |
| HT115: *jxEx4* | 11.1 ± 0.4 | 14 | 70/80 |  |  |  |  |  |  |
| HT115: *nmur-1; jxEx40* | 13.5 ± 0.5 | 16 | 66/80 | **+ 22** | < 0.0001^b^ | < 0.0001^b^ |  |  |  |
| HT115: *nmur-1; jxEx4* | 12.3 ± 0.3 | 14 | 69/80 | + 11 | 0.05^b^ | 0.02^b^ | **- 9** | 0.001^c^ | 0.01^c^ |
| Suppl. Table 3 (Continued) |  |  |  |  |  |  |  |  |  |
| Strain/Treatment | Mean Lifespan  ± SEM (Days) | 75^th^ Percentile (Days) | No. of Animals Observed/  Total Initial Animals | %  Wild type | *P* Value Against Wild type (Logrank) | *P* Value Against Wild type  (Wilcoxon) | % of Specified Groups | *P* Value Against Specified Groups  (Logrank) | *P* Value Against Specified Groups  (Wilcoxon) |
| *E. coli* LPS-dependence |  |  |  |  |  |  |  |  |  |
| Trial 1 - CS180: Wild type | 13.8 ± 0.4 | 16 | 71/80 |  |  |  |  |  |  |
| Trial 1 - CS180: *nmur-1* | 15.2 ± 0.4 | 17 | 65/80 | + 10 | 0.03 | 0.03 |  |  |  |
| Trial 1 - CS2198: Wild type | 13.6 ± 0.4 | 16 | 69/80 |  |  |  | - 1 | 0.73^d^ | 0.54^d^ |
| Trial 1 - CS2198: *nmur-1* | 15.7 ± 0.4 | 19 | 66/80 | **+ 15** | 0.0003 | 0.0009 | + 3 | 0.14^d^ | 0.35^d^ |
| Trial 1 - CS2429: Wild type | - 1.  ± 0.4 | 16 | 72/80 |  |  |  | 0 | 0.97^d^ | 0.84^d^ |
| Trial 1 - CS2429: *nmur-1* | 16.9 ± 0.5 | 19 | 68/80 | **+ 22** | < 0.0001 | < 0.0001 | **+ 11** | 0.001^d^ | 0.007^d^ |
| Trial 1 - CS1861: Wild type | 13.4 ± 0.5 | 17 | 72/80 |  |  |  | - 3 | 0.99^d^ | 0.61^d^ |
| Trial 1 - CS1861: *nmur-1* | 14.5 ± 0.5 | 17 | 63/80 | + 8 | 0.17 | 0.12 | - 5 | 0.56^d^ | 0.51^d^ |
|  |  |  |  |  |  |  |  |  |  |
| Trial 2 - OP50: Wild type | 11.9 ± 0.8 | 17 | 34/40 |  |  |  |  |  |  |
| Trial 2 - OP50: *nmur-1* | 17.5 ± 0.5 | 19 | 31/40 | **+ 47** | < 0.0001 | < 0.0001 |  |  |  |
| Trial 2 - CS180: Wild type | 14.9 ± 0.4 | 17 | 69/80 |  |  |  |  |  |  |
| Trial 2 - CS180: *nmur-1* | 15.4 ± 0.4 | 17 | 64/80 | + 3 | 0.35 | 0.32 |  |  |  |
| Trial 2 - CS2198: Wild type | 12.8 ± 0.3 | 15 | 72/80 |  |  |  | **- 14** | 0.0004^d^ | < 0.0001^d^ |
| Trial 2 - CS2198: *nmur-1* | 15.9 ± 0.4 | 17 | 68/81 | **+ 24** | < 0.0001 | < 0.0001 | + 3 | 0.20^d^ | 0.41^d^ |
| Trial 2 - CS2429: Wild type | 13.2 ± 0.4 | 17 | 69/80 |  |  |  | **- 11** | 0.05^d^ | 0.001^d^ |
| Trial 2 - CS2429: *nmur-1* | 16.4 ± 0.7 | 21 | 52/80 | **+ 24** | 0.0002 | 0.0001 | + 6 | 0.02^d^ | 0.25^d^ |
|  |  |  |  |  |  |  |  |  |  |
| Trial 3 - CS180: Wild type | 14.3 ± 0.4 | 17 | 65/80 |  |  |  |  |  |  |
| Trial 3 - CS180: *nmur-1* | 14.7 ± 0.4 | 17 | 73/80 | + 3 | 0.88 | 0.0.52 |  |  |  |
| Trial 3 - CS2429: Wild type | 12.8 ± 0.4 | 14 | 72/80 |  |  |  | **- 10** | 0.03^d^ | 0.007^d^ |
| Trial 3 - CS2429: *nmur-1* | 15.4 ± 0.5 | 18 | 68/80 | **+ 20** | 0.0001 | < 0.0001 | + 5 | 0.05^d^ | 0.17^d^ |
|  |  |  |  |  |  |  |  |  |  |
|  |  |  |  |  |  |  |  |  |  |
|  |  |  |  |  |  |  |  |  |  |
