# Supplementary material for: A Neuromedin U Receptor Acts with the Sensory System to Modulate Food Type-Dependent Effects on C. elegans Lifespan
Source: PLoS Biol. 2010 May 25;8(5):e1000376. doi: 10.1371/journal.pbio.1000376 (PMC2876044; doi:10.1371/journal.pbio.1000376)
Supplement: Table S4 — Fat storage of wild-type and nmur-1 mutant worms on OP50 and HT115. Fat storage in 1-d-old adults is quantified by labeling the worms with C1-BODIPY-C12 according to Mak et al. [84]. All quantifications are normalized to wild type on OP50 and given as percent ± SEM. Numbers in parentheses indicate the number of worms assayed for each condition. The superscript a indicates p = 0.042 compared to wild type on OP50. (0.03 MB DOC) [file pbio.1000376.s009.doc]

Supplementary Table 4. Fat storage of wild-type and *nmur-1* mutant worms on OP50 and HT115.

| Worm strain | OP50 | HT115 |
| --- | --- | --- |
| Wild type | 100 ± 6 (16) | 72 ± 5^a^ (14) |
| *nmur-1* | 86 ± 11 (16) | 97 ± 9 (15) |
